# SHICEDO: single-cell Hi-C data enhancement with reduced over-smoothing

**DOI:** 10.1093/bioinformatics/btaf575

**Published:** 2025-10-23

**Authors:** Jingong Huang, Rui Ma, Michael Strobel, Yangyang Hu, Tiantian Ye, Tao Jiang, Wenxiu Ma

**Affiliations:** Department of Computer Science and Engineering, University of California Riverside, CA 92521, United States; Department of Statistics, University of California Riverside, CA 92521, United States; Department of Computer Science and Engineering, University of California Riverside, CA 92521, United States; Department of Computer Science and Engineering, University of California Riverside, CA 92521, United States; Department of Statistics, University of California Riverside, CA 92521, United States; Department of Computer Science and Engineering, University of California Riverside, CA 92521, United States; Institute of Integrative Genome Biology, University of California Riverside, CA 92521, United States; Department of Statistics, University of California Riverside, CA 92521, United States; Institute of Integrative Genome Biology, University of California Riverside, CA 92521, United States

## Abstract

**Motivation:**

Single-cell Hi-C (scHi-C) technologies have significantly advanced our understanding of the 3D genome organization. However, scHi-C data are often sparse and noisy, leading to substantial computational challenges in downstream analyses.

**Results:**

In this study, we introduce SHICEDO, a novel deep-learning model specifically designed to enhance scHi-C contact matrices by imputing missing or sparsely captured chromatin contacts through a generative adversarial framework. SHICEDO leverages the unique structural characteristics of scHi-C matrices to derive customized features that enable effective data enhancement. Additionally, the model incorporates a channel-wise attention mechanism to mitigate the over-smoothing issue commonly associated with scHi-C enhancement methods. Through simulations and real-data applications, we demonstrate that SHICEDO outperforms the state-of-the-art methods, achieving superior quantitative and qualitative results. Moreover, SHICEDO enhances key structural features in scHi-C data, thus enabling more precise delineation of chromatin structures such as A/B compartments, TAD-like domains, and chromatin loops.

**Availability and implementation:**

SHICEDO is publicly available at https://github.com/wmalab/SHICEDO.

## 1 Introduction

The three-dimensional (3D) genome architecture is fundamental to the regulation of key biological processes such as gene transcription, DNA replication, and cell division (Misteli 2020). Hi-C techniques (Lieberman-Aiden *et al.* 2009, Duan *et al.* 2010, Rao *et al.* 2014, Ma *et al.* 2015) provide genome-wide mapping of chromatin contacts, shedding light on the principles of 3D genome organization. Analyses of Hi-C contact frequency matrices have unveiled multiple levels of chromatin organization, including active and inactive (A/B) compartments (Lieberman-Aiden *et al.* 2009), topologically associated domains (TADs) (Dixon *et al.* 2012), and chromatin loops (Rao *et al.* 2014).

Recently, the advent of single-cell Hi-C (scHi-C) technologies (Nagano *et al.* 2013, 2017, Ramani *et al.* 2017) has further revolutionized the field by enabling the investigation of 3D genome architecture at the single-cell level, yielding invaluable insights into the variability and dynamics of spatial genome organization of individual cells. However, scHi-C data remain limited to a few cell lines or tissues due to experimental constraints and the high sequencing cost. Furthermore, the presently available scHi-C datasets often suffer from low sequencing depth, substantial sparsity, experimental biases, and noise, all of which pose significant computational challenges for downstream data analyses. Among these challenges, low sequencing depth and sparsity are particularly significant, as they substantially hinder comprehensive analyses of scHi-C data.

Several computational methods, commonly referred to as imputation or data enhancement approaches, have been developed to address this challenge. These methods aim to infer missing or sparsely captured chromatin contacts and computationally increase the effective sequencing depth, thereby reducing sparsity and improving the overall data quality and interpretability of scHi-C data. Due to the inherent sparse nature of scHi-C data, such methods typically do not increase the resolution of scHi-C matrices in the traditional sense, i.e. they do not reduce the size of genomic bins or increase the dimensionality of the contact matrices.

For example, scHiCluster (Zhou *et al.* 2019) used convolution and random walk with restart (RWR) imputation to mitigate data sparsity before downstream clustering. Similarly, SnapHiC (Yu *et al.* 2021) used RWR-based imputation to improve loop detection. However, imputation strategies based on convolution and random walk often rely on local information, which can introduce false positive contacts and lead to over-smoothing, a phenomenon where fine structural details in the imputed matrices are obscured. scHiCcompare (Nguyen *et al.* 2025) proposed a different approach: it incorporated genomic distance decay into its design by using a distance-aware random forest model, grouping chromatin contacts of similar genomic distance for imputation. Although this helps reduce bias from unrelated contacts, it may still contribute to over-smoothing by borrowing information across similar distances without preserving fine-grained structures. Zhang *et al.* (2022) introduced Higashi, a hypergraph-based deep learning method designed to tackle the sparsity issue in scHi-C data. By conceptualizing scHi-C data as a hypergraph, Higashi effectively harnesses global information across cells by aggregating signals from similar cells to enhance shared chromatin features. While Higashi improves scHi-C data quality, its aggregation strategy may also introduce over-smoothing (as depicted in Fig. S1, available as supplementary data at *Bioinformatics* online). Over-smoothed scHi-C matrices often lose fine structural details, which can lead to false identification of chromatin features. Moreover, over-smoothing can inadvertently reduce cell-to-cell variability.

Alternatively, deep learning-based methods have been developed to enhance Hi-C data. These approaches typically adopt a supervised learning framework, in which a downsampled matrix is enhanced to approximate the original high-coverage Hi-C matrix. Several convolutional neural network (CNN) and generative adversarial network (GAN) models have been developed for enhancing bulk Hi-C data, including HiCPlus (Zhang *et al.* 2018), hicGAN (Liu *et al.* 2019), DeepHiC (Hong *et al.* 2020), and EnHiC (Hu and Ma 2021). While originally designed for bulk Hi-C data, these methods can be adapted for sparse scHi-C data. However, such adaptation often results in over-smoothed scHi-C matrices (see Fig. S1, available as supplementary data at *Bioinformatics* online). More recently, ScHiCEDRN (Wang *et al.* 2023) has been developed to enhance scHi-C data by treating it as a one-channel image and using a GAN framework inspired by super-resolution imaging techniques. While promising, this approach occasionally produces image artifacts and tends to predict an excessive number of false positive contacts (see Fig. S1, available as supplementary data at *Bioinformatics* online).

Traditional CNN-based models, though effective in image applications, face unique challenges when applied to inherently sparse scHi-C data. Unlike natural images, scHi-C data are represented as one-channel symmetric matrices, where structural patterns are strongly influenced by genomic distance rather than spatial continuity. This contrasts with the smooth intensity gradients typically observed in images. In this context, standard CNN parameter sharing may fail to capture key features. Moreover, designing convolutional kernels that effectively extract both fine-grained signals and large-scale structural patterns is particularly challenging in the presence of high data sparsity.

To overcome these limitations, we developed SHICEDO, a novel GAN-based deep-learning approach specifically designed for scHi-C data enhancement. SHICEDO incorporates specialized scHi-C feature extraction and channel-wise attention mechanisms, building on our previous work EnHiC (Hu and Ma 2021). It substantially improves the effective coverage and quality of scHi-C data while preserving its unique structural characteristics. We conducted a comprehensive evaluation to assess the accuracy and quality of SHICEDO-enhanced scHi-C data. Our analyses include both pixel-wise and Hi-C-specific similarity measures, as well as performance in key downstream applications. Results consistently show that SHICEDO effectively enhances sparse scHi-C data while maintaining its structural integrity. The capability and effectiveness of SHICEDO are further demonstrated across downstream analyses, including A/B compartment identification, TAD-like domain detection, and single-cell loop detection. Thus, SHICEDO provides an effective and robust solution to scHi-C data sparsity, enabling in-depth exploration of chromatin organization at the single-cell level.

## 2 Materials and methods

### 2.1 Overview of the SHICEDO model architecture

In this work, we introduce SHICEDO, a novel deep-learning model specifically designed to enhance the quality of scHi-C data while mitigating the over-smoothing issue. Built on a GAN framework, SHICEDO’s generator processes sparse or downsampled scHi-C input of different sizes or scales, producing enhanced contact maps as output. Leveraging our previous work on bulk Hi-C enhancement, EnHiC (Hu and Ma 2021), we have incorporated and refined its rank-one feature extraction and reconstruction techniques, along with our newly developed feature refinement modules, into the SHICEDO framework.

**Figure 1.**
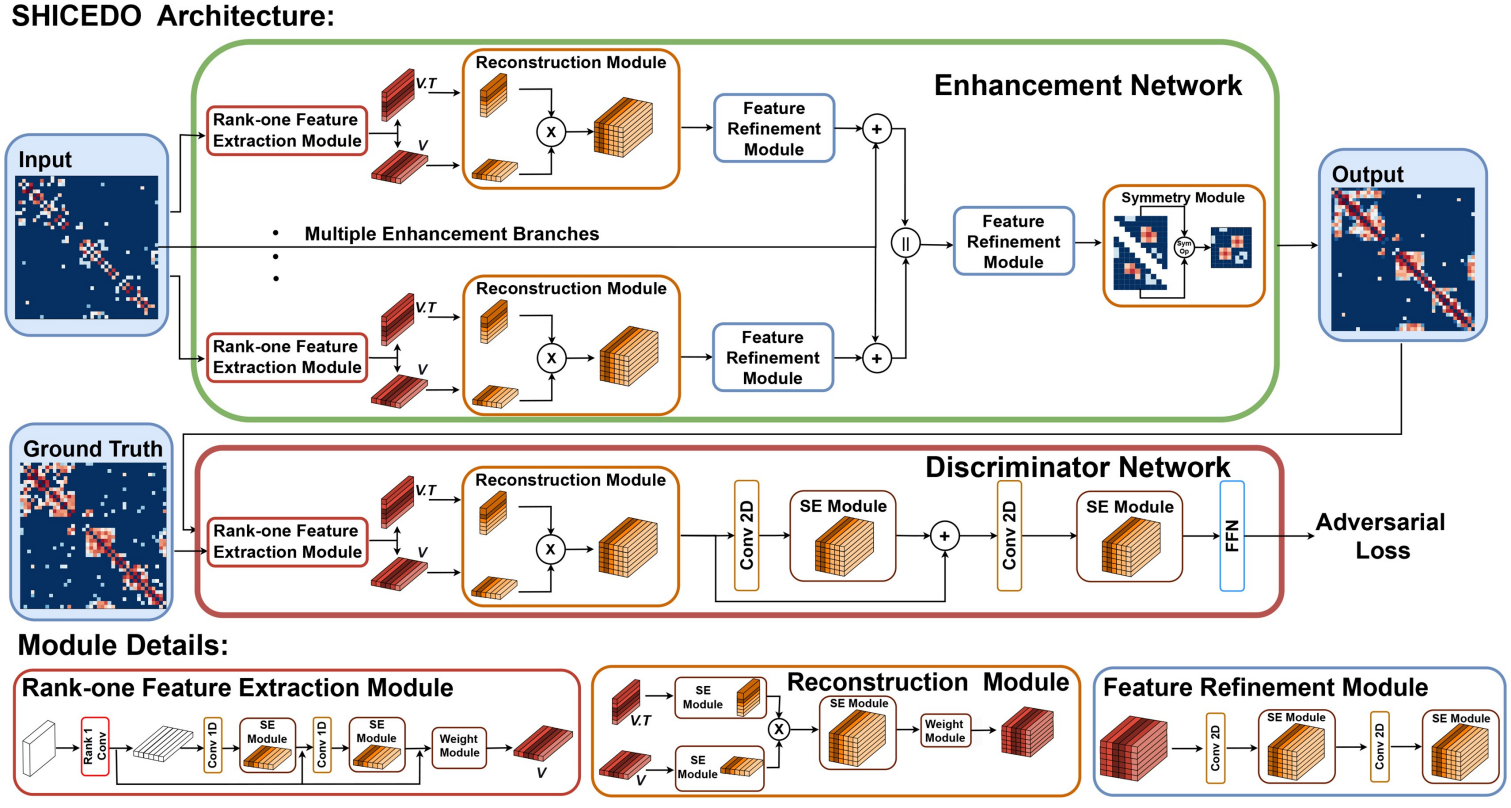
Schematic illustration of the SHICEDO model for scHi-C data enhancement. The model architecture consists of a generator (Enhancement Network) and a discriminator (Discriminator Network) built on a GAN framework. Symbols and abbreviations are explained in Note 2.1, available as supplementary data at *Bioinformatics* online.

As illustrated in Fig. 1, the GAN model in SHICEDO consists of two key components: the generator (Enhancement Network) and the discriminator (Discriminator Network). The generator transforms sparse, low-coverage scHi-C matrices into enhanced matrices with denser and more complete chromatin contact patterns, while the discriminator distinguishes between enhanced and authentic scHi-C matrices. This adversarial interplay drives the model to continuously improve, yielding high-quality, biologically meaningful enhancements of scHi-C data. Further details on the SHICEDO model are provided in Notes 2.1–2.7, available as supplementary data at *Bioinformatics* online.

SHICEDO’s generator is designed with flexibility, supporting multiple input branches that can process submatrices of different sizes or resolution scales (see Note 2.6, available as supplementary data at *Bioinformatics* online). This design allows users to select multi-size input for broader contextual information or multi-scale input for capturing both fine-grained and large-scale structural features. Guided by our ablation study (see Note 2.8, available as supplementary data at *Bioinformatics* online), we adopted a dual-branch configuration for the datasets analyzed in this study. In this setup, the two branches process sparse, low-coverage submatrices of different sizes, enabling the extraction of both central and boundary features. This dual-branch strategy also effectively reduces edge artifacts when merging submatrices back into complete scHi-C matrices.

### 2.2 Key design strategies of SHICEDO

The SHICEDO model architecture incorporates two specialized design components, each tailored to address the unique challenges of scHi-C data enhancement. The first, rank-one feature extraction, improves the capture of structural information from sparse scHi-C matrices. The second, channel-wise feature selection, mitigates the over-smoothing problem that commonly arises in existing Hi-C enhancement methods.

#### 2.2.1 Rank-one feature extraction

An integral part of SHICEDO’s feature selection strategy is the adaptation of the rank-one feature extraction concept, first introduced in our earlier bulk Hi-C enhancement work, EnHiC (Hu and Ma 2021). In SHICEDO, this module has been refined to better capture key structural features in sparse scHi-C data.

For an input matrix of size n×n, the rank-one module applies a convolution kernel of size n×1. This design is well-suited to sparse, symmetric scHi-C matrices, where conventional square kernels may fail to capture informative patterns. The rank-one kernel promotes parameter sharing across genomic bins, achieving more efficient feature extraction. Because sparse scHi-C matrices exhibit heterogeneous patterns within local regions, this approach helps preserve meaningful structural details that might otherwise be overlooked by traditional 2D convolutional layers.

Compared to our previous bulk Hi-C implementation, this refined rank-one module integrates two additional 1D convolutional layers for more precise feature extraction. Additionally, we incorporated a squeeze-and-excitation (SE) module (Hu *et al.* 2018) and skip connections to improve information flow and mitigate information loss. Furthermore, the reconstruction module derives feature tensors through the outer product of rank-one tensors, ensuring that the enhanced matrix preserves the intrinsic symmetry characteristic of Hi-C matrices.

Further details on the design and implementation of rank-one feature extraction are provided in Notes 2.3 and 2.4, available as supplementary data at *Bioinformatics* online.

#### 2.2.2 Channel-wise feature selection

Another key component of SHICEDO is the channel-wise attention mechanism (Hu *et al.* 2018), specifically designed to enhance feature selection across multiple channels of the feature tensor. Although each scHi-C submatrix enters the network as a single-channel contact map, subsequent convolutions expand this into many feature channels. Not all channels are equally informative: some capture biologically meaningful structures, while others encode noise.

Channel-wise attention adaptively weighs these channels, amplifying useful features while suppressing uninformative ones. This is especially important for sparse scHi-C data, where conventional CNN channel summation often produces over-smoothed results that obscure fine structural details. Over-smoothing arises not from failure to recognize structures but from indiscriminate mixing of pixels, which blurs local interactions and reduces cell-to-cell variability.

To address this, SHICEDO integrates SE modules throughout the architecture. Each SE module applies pooling and a small two-layer perceptron to generate learnable channel weights, dynamically adjusting feature contributions. By focusing on biologically relevant patterns and filtering noise, SHICEDO preserves fine structural details, improves enhancement quality, and mitigates over-smoothing.

### 2.3 scHi-C datasets and preprocessing

We applied SHICEDO to four publicly available scHi-C datasets: the human brain prefrontal cortex dataset (Lee *et al.* 2019), the developing mouse embryo dataset (Liu *et al.* 2023), the mouse embryonic stem (ES) cell dataset (Nagano *et al.* 2017), and the developing mouse brain Dip-C dataset (Tan *et al.* 2021) (Table S1, available as supplementary data at *Bioinformatics* online). After preprocessing, we generated scHi-C matrices at three resolutions (1 Mb, 100 kb, and 50 kb) with five downsampling ratios (2×, 4×, 9×, 16×, and 36×).

Matrices at suitable resolutions and chromatin contacts within predefined genomic-distance thresholds were used for downstream analyses, including A/B compartment identification, TAD-like domain detection, and chromatin loop calling (Table S2, available as supplementary data at *Bioinformatics* online). Additional details on data preprocessing steps are described in Note 2.9, available as supplementary data at *Bioinformatics* online, and training and prediction configurations are provided in Note 2.10, available as supplementary data at *Bioinformatics* online.

## 3 Results

### 3.1 SHICEDO faithfully enhances scHi-C matrices

To evaluate the performance of SHICEDO in scHi-C data enhancement, we conducted a comprehensive assessment using both pixel-wise metrics and Hi-C-specific similarity measures. We compared SHICEDO against two state-of-the-art scHi-C enhancement methods, ScHiCEDRN (Wang *et al.* 2023) and Higashi (Zhang *et al.* 2022), as well as two bulk Hi-C enhancement models, EnHiC (Hu and Ma 2021) and DeepHiC (Hong *et al.* 2020). This evaluation aimed to assess SHICEDO’s ability to accurately enhance sparse scHi-C matrices while preserving their structural properties.

For pixel-wise evaluations, we used two key metrics: mean absolute error (MAE) and macro F1 score. MAE quantifies pixel-level accuracy, with lower values indicating closer agreement with the ground truth, the original high-coverage matrices. The macro F1 score provides a balanced measure of precision and recall, effectively identifying potential over-smoothing effects. To compute it, sparse scHi-C matrices were binarized, where the smallest non-zero value in the ground truth served as the threshold, and all values above this threshold in the enhanced matrix were treated as positives. The macro F1 score ranges from 0 to 1, with higher values indicating better performance.

**Figure 2.**
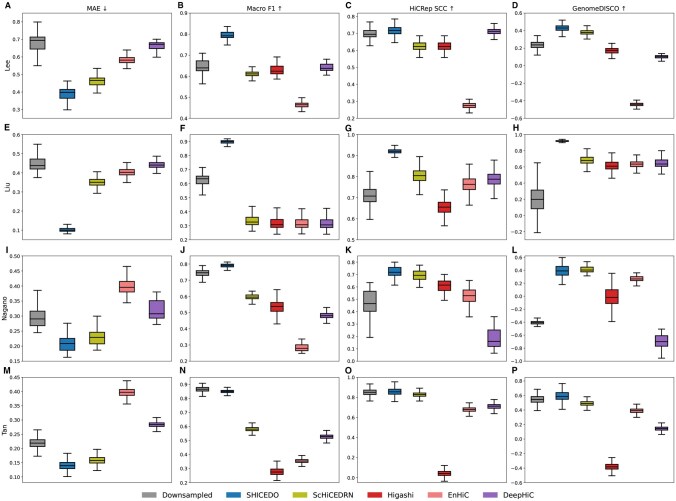
Pixel-wise and Hi-C-specific similarity evaluation of enhanced scHi-C data. Evaluation results are presented for four scHi-C datasets at 1-Mb resolution: (A–D) Lee *et al.* (36× downsampling), (E–H) Liu *et al.* (16× downsampling), (I–L) Nagano *et al.* (9× downsampling), and (M–P) Tan *et al.* (4× downsampling). For each dataset, two pixel-wise metrics, mean absolute error (MAE) and macro F1 score, were computed by comparing downsampled sparse input and enhanced scHi-C submatrices against the ground truth. Note that MAE values for Higashi are not plotted, as the method does not specifically optimize for pixel-wise predictions. Additionally, two Hi-C-specific similarity measures, stratum-adjusted correlation coefficients (SCC) from HiCRep and GenomeDISCO scores, were calculated using chromosome-wide matrices.

We first evaluated the scHi-C dataset from human prefrontal cortex cells by Lee *et al.* at 1-Mb resolution using 36× downsampled sparse input matrices. The enhanced matrices were compared against the ground truth (i.e. the original high-coverage matrices). As shown in Fig. 2, SHICEDO consistently outperformed baseline methods in both MAE and macro F1 score (Fig. 2A and B). For MAE, SHICEDO achieved the lowest score of 0.3908, surpassing the best-performing baseline method, ScHiCEDRN (*P*-value $=2.5\times 10^{-100}$, *t*-test). For macro F1 score, SHICEDO obtained the highest score of 0.7951, significantly outperforming the best-performing baseline, DeepHiC (*P*-value $=2.1\times 10^{-247}$, *t*-test). Note that the high macro F1 score in downsampled sparse input is largely due to the inherent sparse nature of scHi-C matrices, where most pixels are zeros. Downsampling reduces the values of high-count pixels but rarely sets them to zero, which increases precision and leads to an inflated macro F1 score. In contrast, other baseline methods tended to over-smooth the matrices, introducing false-positive pixels that lowered precision and, consequently, macro F1 scores. Therefore, the macro F1 score serves as a reliable indicator of over-smoothing, and our results confirm that SHICEDO effectively mitigates this issue.

In addition to pixel-wise evaluations, we assessed performance using two Hi-C-specific similarity measures: HiCRep (Yang *et al.* 2017, Lin *et al.* 2021) and GenomeDISCO (Ursu *et al.* 2018). These metrics further validated SHICEDO’s effectiveness in preserving structural features in scHi-C data. HiCRep accounts for Hi-C-specific biases and noise by applying smoothing and stratifying contact counts based on genomic distance. The resulting stratum-adjusted correlation coefficient (SCC) ranges from −1 to 1, with 1 indicating identical Hi-C matrices. On the other hand, GenomeDISCO evaluates Hi-C matrix similarity by modeling it as a graph, producing a similarity score also ranging from −1 to 1, with 1 reflecting perfect agreement.

Figure 2C and D presents the average HiCRep SCC and GenomeDISCO values of merged intra-chromosomal matrices across individual cells in the Lee *et al.* dataset. Notably, SHICEDO achieved the highest HiCRep SCC value of 0.7175, outperforming DeepHiC (*P*-value $=3.5\times 10^{-3}$, *t*-test). Additionally, SHICEDO obtained the highest GenomeDISCO scores of 0.4245, significantly surpassing ScHiCEDRN (*P*-value $=5.9\times 10^{-42}$, *t*-test).

Furthermore, we evaluated SHICEDO on three additional scHi-C datasets: developing mouse embryonic cells from Liu *et al.* (1-Mb resolution, 16× downsampling), mouse ES cells by Nagano *et al.* (1-Mb resolution, 9× downsampling), and developing mouse brain cells by Tan *et al.* (1-Mb resolution, 4× downsampling). These datasets span different scHi-C protocols, cell numbers, and sequencing depths. Our results demonstrated that SHICEDO consistently outperformed baseline methods across all datasets in terms of MAE, macro F1 scores, and HiCRep SCC values. Additionally, SHICEDO achieved the highest GenomeDISCO scores on the Liu *et al.* and Tan *et al.* datasets, and performed comparably to ScHiCEDRN in GenomeDISCO score on the Nagano *et al.* dataset (Fig. 2). Collectively, these results confirm that SHICEDO robustly enhances scHi-C data quality while mitigating over-smoothing.

To contextualize SHICEDO’s performance, we further compared it against several lightweight imputation methods, which are computationally efficient but less sophisticated. Unsurprisingly, SHICEDO consistently achieved the best performance across all evaluation metrics, whereas the lightweight baselines showed mixed or degraded performance depending on sparsity (see Note 2.11, available as supplementary data at *Bioinformatics* online). Taken together, our results demonstrate that SHICEDO not only restores fine-scale chromatin contacts but also preserves global chromatin organization more effectively than both state-of-the-art deep learning models and classical lightweight methods.

### 3.2 SHICEDO effectively enhances scHi-C data across different cell types and species

The Lee *et al.* dataset comprises 14 distinct cell types from the human prefrontal cortex. To evaluate SHICEDO’s adaptability to new data, we trained the model on six selected cell types: ODC, Astro, OPC, Sst, Endo, and Vip, and validated on MP and L6 cells. Testing was subsequently conducted on the remaining six cell types: MG, Ndnf, Pvalb, L4, L5, and L23. The training, validation, and test set consisted of approximately 75%, 5%, and 20% of the cells, respectively.

Following enhancement with SHICEDO, the model achieved an MAE value of 0.3074, a macro F1 score of 0.8357, a HiCRep SCC of 0.7829, and a GenomeDISCO score of 0.4856 on the test data. For comparison, when trained and tested on the same Lee *et al.* dataset without distinguishing cells by their types, under identical conditions (i.e. 1-Mb resolution and a 16× downsampling ratio.), SHICEDO yielded an MAE value of 0.3164, a macro F1 score of 0.8304, a HiCRep SCC of 0.8194, and a GenomeDISCO score of 0.5305. While MAE and macro F1 scores showed slight improvement, HiCRep SCC and GenomeDISCO scores exhibit minor decreases. Overall, SHICEDO’s performance remained within the same range across these two experimental settings, indicating its robustness in handling diverse cell types without requiring specialized retraining.

To further evaluate the generalizability of SHICEDO across species, we trained the model on scHi-C data from human brain prefrontal cortex cells by Lee *et al.* at 1-Mb resolution with a 16× downsampling ratio, and tested it on mouse ES cells from Nagano *et al.* at 1-Mb resolution with 9× downsampling ratio.

On the mouse ES cell dataset, SHICEDO achieved an MAE of 0.2265, representing a 25.02% reduction compared to downsampled sparse input. It also yielded a macro F1 score of 0.8004, reflecting an 8.85% improvement, a HiCRep SCC of 0.6632, marking a 26.28% increase, and a GenomeDISCO score of 0.2686, showing a 166.45% improvement over the downsampled sparse input data. In comparison, when trained and tested on the same Nagano *et al.* dataset under identical conditions (i.e. 1-Mb resolution, 9× downsampling), SHICEDO achieved an MAE of 0.2110, a macro F1 score of 0.7854, a HiCRep SCC score of 0.7137, and a GenomeDISCO score of 0.3953. Notably, when trained and tested on different species, SHICEDO exhibited minor decreases in MAE, HiCRep SCC, and GenomeDISCO scores but showed an increase in macro F1 score.

These results demonstrate that SHICEDO maintains strong performance across different cell types and exhibits only a slight performance drop across species. This indicates that the model effectively learns transferable chromatin structure features. Such generalizability is crucial, as it allows SHICEDO to enhance scHi-C data quality across diverse cell types and species without requiring extensive retraining.

### 3.3 SHICEDO facilitates robust identification of single-cell A/B compartments

In this section, we evaluate SHICEDO’s ability to enhance scHi-C data and improve the detection of single-cell A/B compartments at 1-Mb resolution. We applied SHICEDO and baseline methods to enhance the 16× downsampled scHi-C matrices from the Lee *et al.* dataset, followed by A/B compartment analysis on each single cell, adopting Higashi’s algorithm (Zhang *et al.* 2022) for identifying single-cell A/B compartments.

To assess the effectiveness of the enhancement methods, we calculated the precision, recall, and macro F1 scores of single-cell A/B compartment assignments obtained from downsampled sparse input and enhanced scHi-C matrices, using the ground truth as reference. A prediction was considered correct if the assigned compartment (A or B) matched the true label at a genomic bin. Given the inherent sparsity of scHi-C data, we incorporated a flexible bin-shift threshold, allowing small positional offsets to still be treated as correct matches.

**Figure 3.**
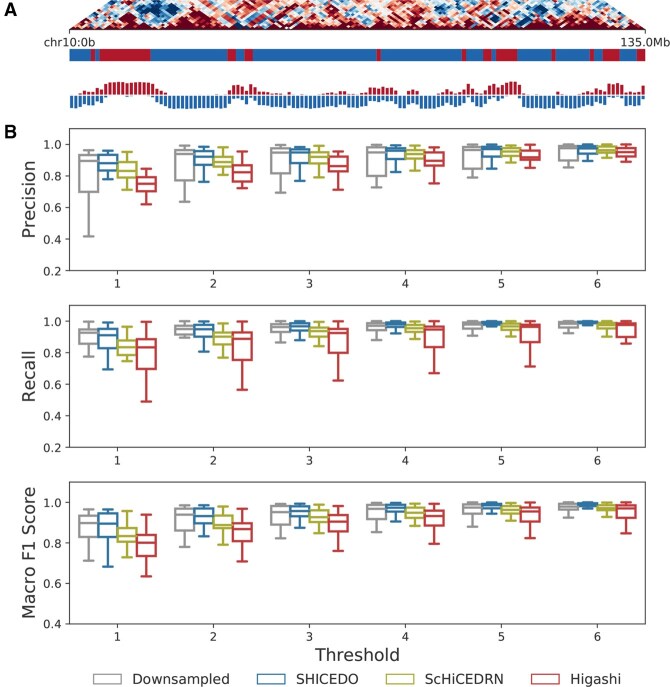
Identification of A/B compartments in enhanced scHi-C data using the Lee *et al.* dataset. (A) Pseudo-bulk Hi-C heatmap, as well as pseudo-bulk and single-cell A/B compartment profiles, are displayed for SHICEDO-enhanced scHi-C matrices on chromosome 10. The pseudo-bulk Hi-C heatmap illustrates the correlation of interaction profiles between pairs of 1-Mb genomic bins; the horizontal barcode represents A/B compartments determined by pseudo-bulk Hi-C, colored in blue and red; the vertical barplot displays the number of cells assigned to A or B compartments at each corresponding genomic bin. (B) Precision, recall, and macro F1 score evaluations for single-cell A/B compartment calls on downsampled sparse input and enhanced scHi-C matrices, compared to the ground truth. Each genomic bin is treated as an independent observation. Predictions and true labels are considered matching if their genomic distance difference falls within a specified threshold (ranging from 1 to 6 bins). Results are based on 1-Mb resolution data with 36× downsampling.

As shown in Fig. 3, Fig. S2, available as supplementary data at *Bioinformatics* online, and Table S3, available as supplementary data at *Bioinformatics* online, SHICEDO-enhanced scHi-C data consistently demonstrated highly accurate single-cell A/B compartment identification, evident in both visual representation and numerical evaluations. Figure 3A highlights the strong concordance between single-cell compartment labels and those derived from pseudo-bulk data aggregated across all cells, confirming the reliability of the single-cell A/B compartment calling. When comparing A/B compartments identified from downsampled sparse input with the ground truth, we observed that the identification of higher-order genome structures, such as A/B compartments, remains relatively robust despite data sparsity (Fig. S2, available as supplementary data at *Bioinformatics* online). Yet, SHICEDO-enhanced scHi-C matrices outperformed all tested methods, particularly at relaxed thresholds, as indicated by comparable or higher precision, recall and macro F1 scores, along with reduced cell-to-cell variations (Fig. 3B and Table S3, available as supplementary data at *Bioinformatics* online). In contrast, ScHiCEDRN and Higashi exhibited a tendency to over-smooth the data, resulting in decreased performance.

These results confirm SHICEDO’s ability to accurately preserve active and inactive states of genomic bin states across various cells while effectively enhancing sparse scHi-C data into more detailed and biologically meaningful representations.

### 3.4 SHICEDO enhances accurate detection of TAD-like domain boundaries

To further assess SHICEDO’s capability to promote accurate detection of TAD-like domain boundaries in scHi-C data, we analyzed scHi-C matrices from the Lee *et al.* dataset at 100-kb resolution with 9× downsampling. After enhancement, we identified TAD-like domains using an insulation score based procedure used by Higashi (Zhang *et al.* 2022). Consistent with previous approaches such as EnHiC and ScHiCEDRN, our TAD-like domain detection focused on chromatin contacts within a 2-Mb genomic distance.

**Figure 4.**
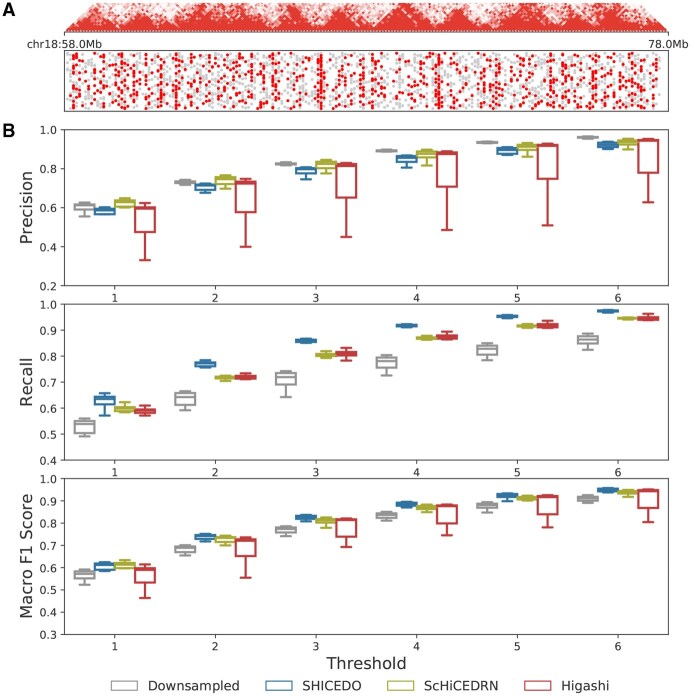
Detection of TAD-like domains in enhanced scHi-C data from the Lee *et al.* dataset. (A) Snapshot of single-cell TAD-like domain boundaries detected based on SHICEDO-enhanced scHi-C matrices within chr18:58–78 Mb is shown in dot plots, one line per cell. TAD structures on the pseudo-bulk heatmap are displayed at the top as a reference. Red dots indicate the boundaries that overlap with the ground truth; while non-overlapping boundaries are shown in gray. (B) Precision, recall, and macro F1 scores of TAD-like domain boundaries detected from downsampled sparse input and enhanced scHi-C data, compared to the ground truth. Two boundaries are considered overlapping if their bin distance falls within the specified threshold, ranging from 1 to 6 bins. Results are based on 100-kb resolution data with 9× downsampling.

Figure 4A and Fig. S3, available as supplementary data at *Bioinformatics* online, illustrate single-cell TAD-like boundaries detected from the ground truth, downsampled sparse input, and enhanced scHi-C matrices. The substantial variability of single-cell domain boundaries across cells highlights the high heterogeneity of single-cell TAD structures. Given this variability, we applied a flexible threshold allowing small bin shifts to determine matching boundaries between enhanced matrices and the ground truth. Figure 4B and Table S4, available as supplementary data at *Bioinformatics* online, present the precision, recall, and macro F1 score under this relaxed criterion.

Compared to downsampled sparse input and baseline methods, SHICEDO achieved a distinct improvement in recall. Since domain boundary bins are significantly outnumbered by non-boundary bins, the dataset is highly imbalanced, explaining the higher precision observed in downsampled data. However, even against baseline methods, SHICEDO maintained comparable precision while consistently achieving the highest macro F1 scores across all thresholds, demonstrating its superior overall performance. Furthermore, comparing results across multiple thresholds allows us to assess how well SHICEDO preserves structural details as noisy data points are removed. As the threshold value increased, evaluation scores of baseline methods approached those of SHICEDO, highlighting the impact of over-smoothing in these baseline methods.

In summary, these results provide compelling evidence that SHICEDO promotes reliable and robust detection of TAD-like domains in single cells.

### 3.5 SHICEDO-enhanced scHi-C matrices facilitate fine-scale loop detection

To evaluate SHICEDO’s ability to preserve fine-scale structural features, we assessed its performance in chromatin loop detection using the Lee *et al.* dataset. The 50-kb resolution scHi-C matrices were downsampled by a factor of 2, and chromatin loops were identified in the ground truth, downsampled sparse input, and SHICEDO-enhanced matrices using the state-of-the-art loop caller SnapHiC (Yu *et al.* 2021). Following SnapHiC’s recommendations, we restricted the analysis to chromatin contacts within a genomic distance of 1 Mb.

**Figure 5.**
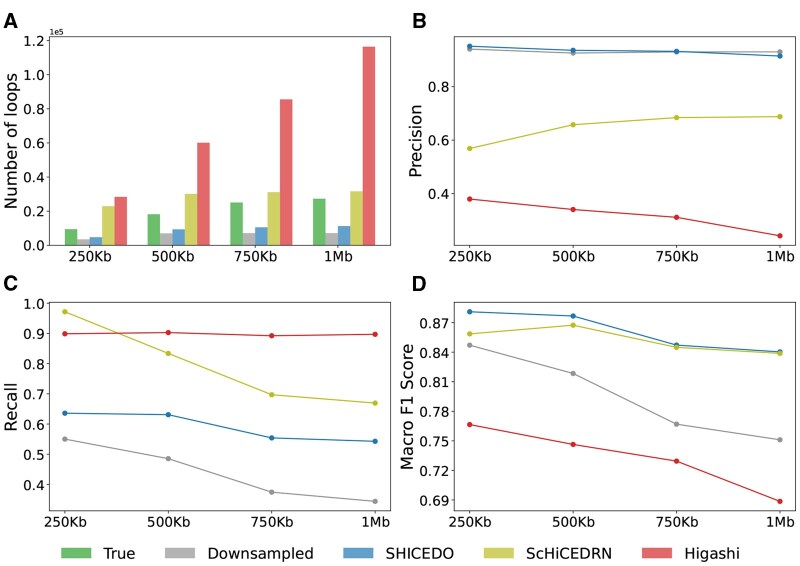
Chromatin loops detected in enhanced scHi-C data from the Lee *et al.* dataset. (A) Total number of detected loops across different genomic distance ranges. (B) Precision, (C) recall, and (D) macro F1 score of loops detected from downsampled sparse input and enhanced scHi-C matrices, compared to the ground truth data. The *X*-axis represents the maximum genomic distance considered for loop detection. A single-pixel shift was allowed while categorizing identical loops. Results are based on 50-kb resolution data with 2× downsampling.

We conducted four sets of experiments to detect chromatin loops within genomic distances of 250 kb, 500 kb, 750 kb, and 1 Mb using SnapHiC. SHICEDO-enhanced scHi-C matrices produced fewer detected chromatin loops overall (Fig. 5A), but the majority of them (e.g. 2750 out of 4744 loops within a 250-kb genomic distance) perfectly overlapped with the ground truth, without a single pixel shift. In contrast, baseline methods ScHiCEDRN and Higashi detected substantially more chromatin loops than the number of true loops across all genomic distance ranges, primarily due to over-smoothing (Fig. 5A). This discrepancy was especially pronounced at the 1-Mb genomic distance range, where the number of loops detected from Higashi-enhanced scHi-C data exceeded the true number by more than 4-fold. Such over-smoothing generated an excessive number of false-positive pixels, which were incorrectly identified as chromatin loops, reducing the reliability of single-cell loop detection. Visual examples of detected chromatin loops in Fig. S4, available as supplementary data at *Bioinformatics* online, illustrate the impact of over-smoothed scHi-C data on loop detection.

To quantify loop detection performance, we computed precision, recall, and macro F1 scores. As shown in Fig. 5B, SHICEDO achieved consistently high precision (∼0.93) across all four genomic distance ranges, while capturing more loops as the genomic distance threshold increases. Figure 5C shows that ScHiCEDRN and Higashi exhibited high recall due to excessive loop predictions. Lastly, as reflected in Fig. 5D, SHICEDO attained the highest macro F1 scores across all thresholds, particularly for loops detected at shorter genomic distances, demonstrating its balanced performance of precision and recall.

In summary, SHICEDO effectively mitigates over-smoothing, leading to more reliable loop detection and facilitating the identification of fine-scale chromatin features in scHi-C data.

## 4 Conclusions and discussion

In this study, we introduced SHICEDO, a GAN-based deep learning approach specifically designed to enhance scHi-C data. SHICEDO improves the effective quality and coverage of scHi-C matrices while preserving structural features and mitigating over-smoothing. Building upon our previous work EnHiC (Hu and Ma 2021), the model integrated refined rank-one feature extraction and reconstruction modules, and further optimizes channel-wise information extraction through Squeeze-and-Excitation Networks (Hu *et al.* 2018).

We rigorously evaluated SHICEDO’s performance on publicly available scHi-C datasets generated from multiple experimental protocols and technologies, spanning different resolutions and sparsity levels to demonstrate its robustness. To comprehensively assess its ability to preserve structural information, we performed a series of multi-scale downstream analyses, including A/B compartment analysis, TAD-like domain detection, and loop detection. Compared with state-of-the-art methods, SHICEDO consistently outperformed existing models, demonstrating its capability to enhance scHi-C matrices while preserving cell-to-cell structural variability and mitigating over-smoothing.

Potential directions for future research include leveraging multi-resolution scHi-C data to achieve a more comprehensive understanding of both local and global chromatin structures, thereby improving prediction accuracy. The main challenge lies in designing model architectures that can effectively handle such complexity. Our current model framework already supports multi-scale input, laying the groundwork for future explorations. Another promising strategy is to directly incorporate global Hi-C information rather than relying solely on local submatrices, which would require innovative computational strategies for efficiently integrating diverse data sources. Furthermore, as scHi-C technologies continue to evolve and datasets expand rapidly in scale and quality, the scalability of current enhancement methods remains an open question. Exploring or improving the scalability of SHICEDO to handle much larger and higher-quality datasets will be critical to ensure its long-term applicability. Together, these future directions hold promise for advancing scHi-C data enhancement methods, enabling further improvements in data quality, recovery of structural details, and broader applicability in 3D genome research.

## Supplementary Material

btaf575_Supplementary_Data

## Data Availability

The SHICEDO source code, together with detailed documentation, is publicly available at https://github.com/wmalab/SHICEDO under the MIT license (DOI: 10.5281/zenodo.17069263).
