# Supplementary material for: SHICEDO: single-cell Hi-C data enhancement with reduced over-smoothing
Source: Bioinformatics. 2025 Oct 23;41(12):btaf575. doi: 10.1093/bioinformatics/btaf575 (PMC12668774; doi:10.1093/bioinformatics/btaf575)
Supplement: btaf575_Supplementary_Data [file btaf575_supplementary_data.pdf]

# Supplementary Materials for “SHICEDO: Single-cell Hi-C Data Enhancement with Reduced Over-smoothing”

Jingong Huang[1], Rui Ma[2], Michael Strobel[1], Yangyang Hu[1], Tiantian Ye[2], Tao Jiang[1,3*], and Wenxiu Ma[2,3*]

[1] Department of Computer Science and Engineering, University of California, Riverside, CA 92521, USA
[2] Department of Statistics, University of California, Riverside, CA 92521, USA
[3] Institute of Integrative Genome Biology, University of California, Riverside, CA 92521, USA

*To whom correspondence should be addressed. Email: jiang@cs.ucr.edu (T.J.), wenxiu.ma@ucr.edu (W.M.)

# 1 Supplementary Figures and Tables

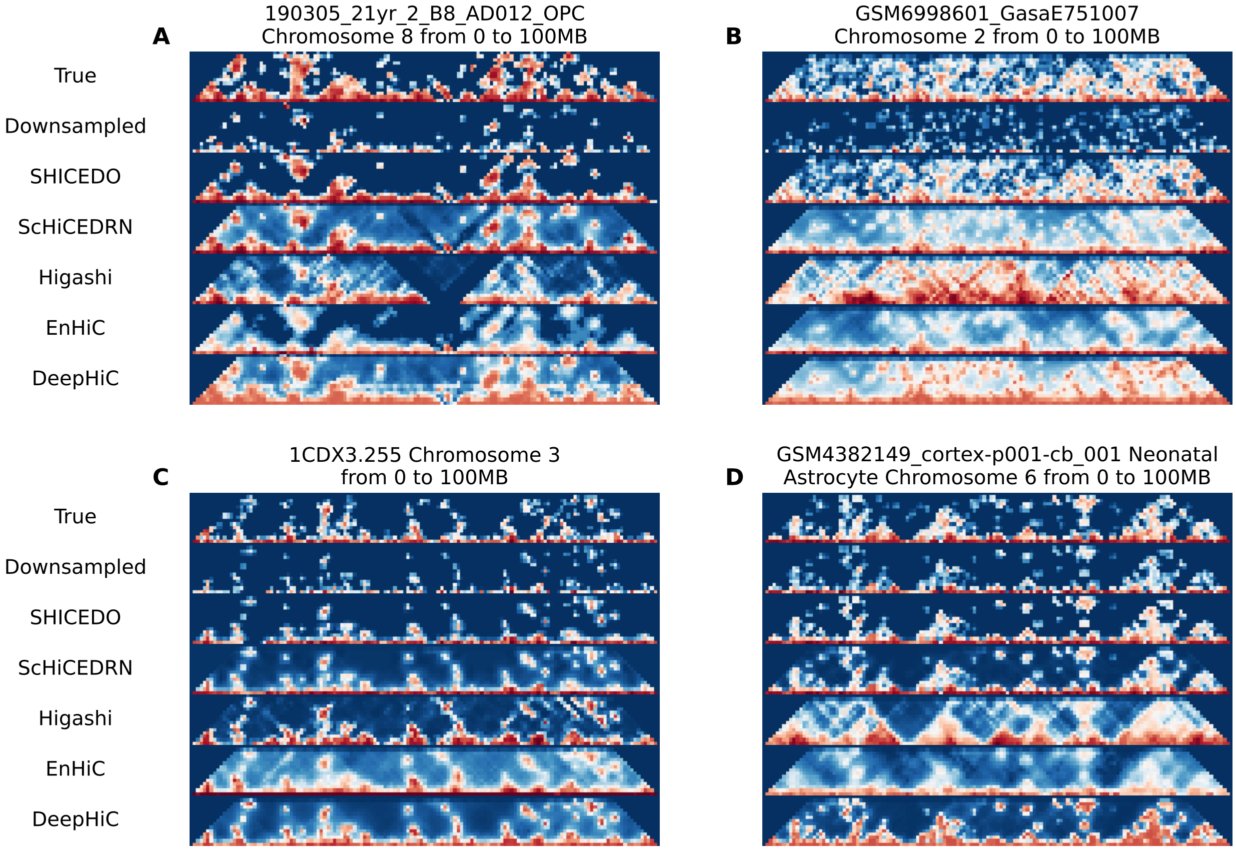


**Supplementary Figure S1**: **Examples of enhanced scHi-C matrices.** Heatmaps display the original high-coverage (True), downsampled sparse input (Downsampled), and enhanced matrices by SHICEDO, ScHiCEDRN, Higashi, EnHiC, and DeepHiC for four genomic regions: (A) Chromosome 8 (0–100 Mb, cell 190305 21yr 2 A11 AD012 L23, Lee *et al.*, 1-Mb resolution, 36× downsampling), (B) Chromosome 2 (0–100 Mb, cell GSM6998601 GasaE751007, Liu *et al.*, 1-Mb resolution, 16× downsampling), (C) Chromosome 3 (0–100 Mb, cell 1CDX3.255, Nagano *et al.*, 1-Mb resolution, 9× downsampling), and (D) Chromosome 6 (0–100 Mb, cell GSM4382149 cortex p001 cb 001 Neonatal Astrocyte, Tan *et al.*, 1-Mb resolution, 4× downsampling).

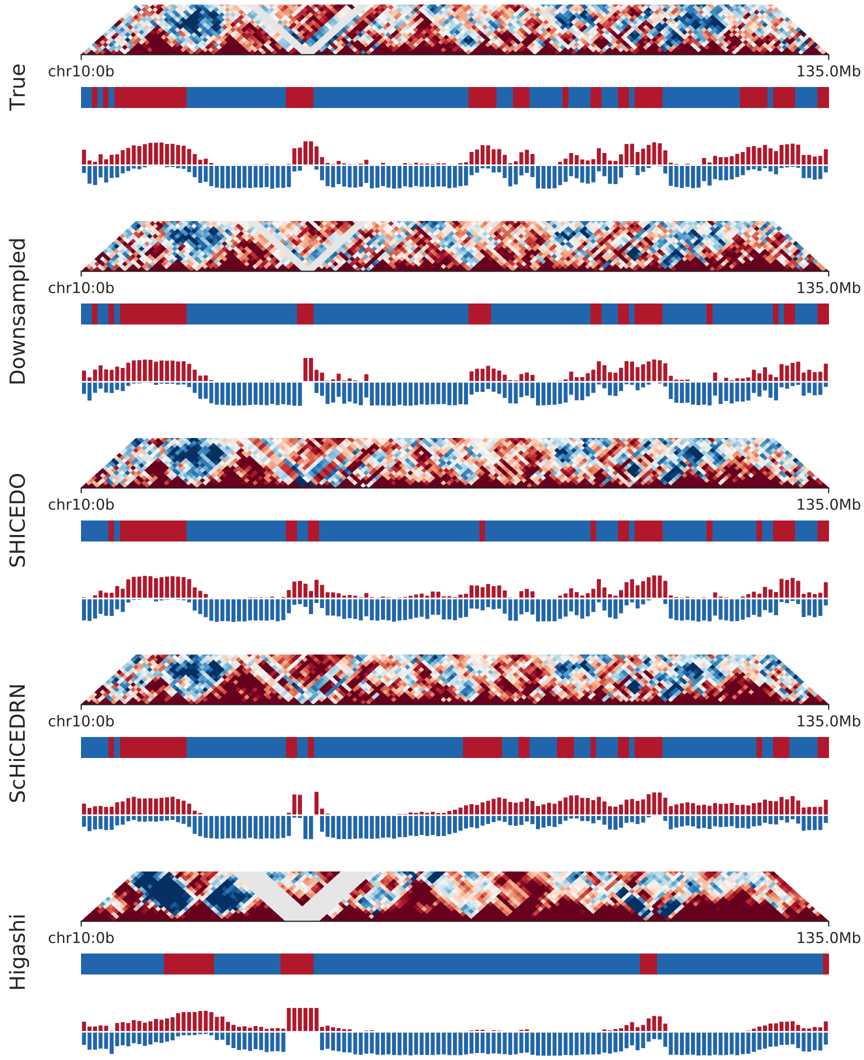


**Supplementary Figure S2**: **Identification of A/B compartments in enhanced scHi-C data from the Lee *et al.* dataset.** Pseudo-bulk Hi-C heatmaps and both pseudo-bulk and single-cell A/B compartment annotations are shown. The five rows represent the ground truth, downsampled sparse input, SHICEDO-enhanced, ScHiCEDRN-enhanced, and Higashi-enhanced scHi-C matrices (from top to bottom) on chromosome 10. A/B compartments were inferred based on the sign of the first component (PC1) computed using FAN-C [1]. The pseudo-bulk Hi-C heatmap shows the correlation (red: 1, blue: -1) of interaction profiles between pairs of 1-Mb genomic bins. The horizontal barcode represents the A/B compartment labels determined by pseudo-bulk Hi-C, colored in blue and red. The vertical barplot displays the number of single cells assigned as A or B compartments at each genomic bins. Results are based on 1-Mb resolution data with 36× downsampling.

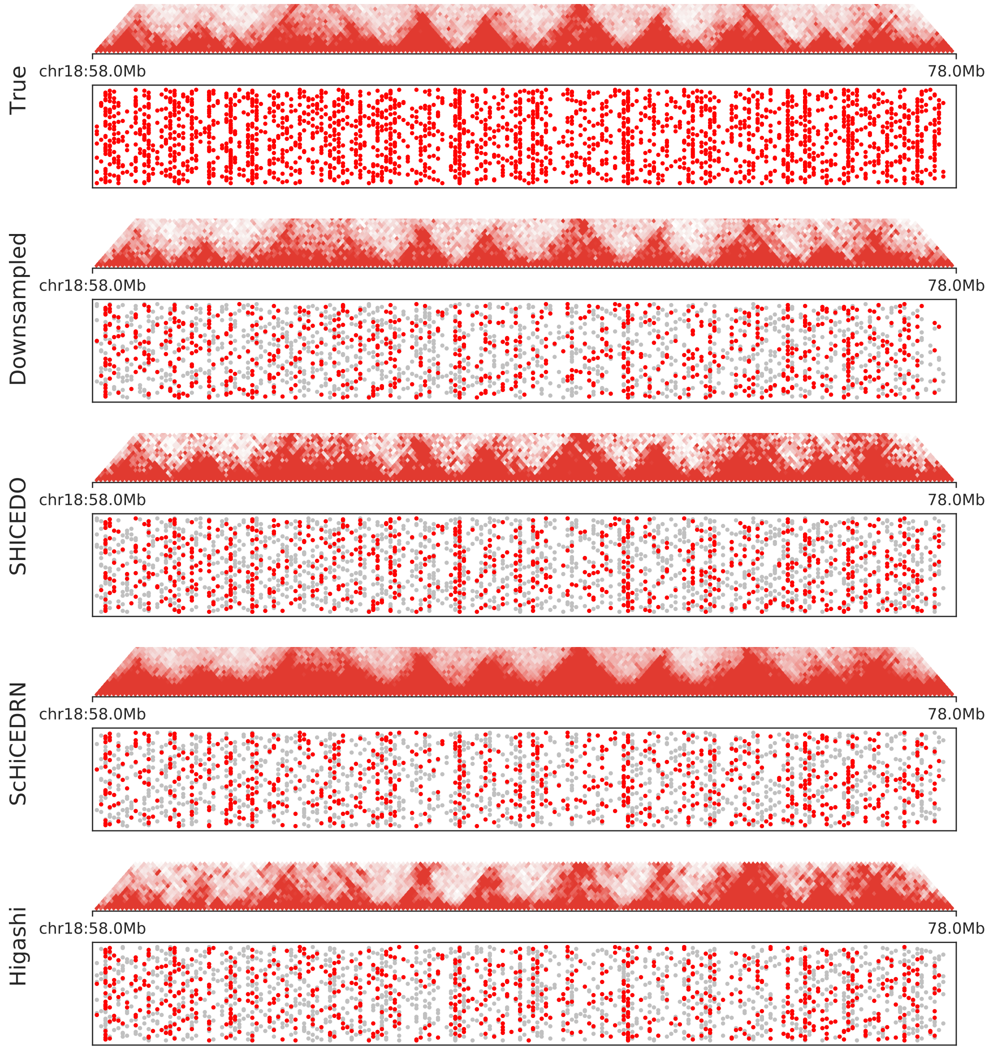


**Supplementary Figure S3**: **Detection of TAD-like domains in enhanced scHi-C data from the Lee *et al.* dataset.** The five rows represent the ground truth, downsampled sparse input, SHICEDO-enhanced, ScHiCEDRN-enhanced, and Higashi- enhanced scHi-C matrices (from top to bottom) on chromosome 18, 58–78 Mb. TAD structures on pseudo-bulk Hi-C heatmap are displayed on top as a reference. Single-cell TAD-like domain boundariesare shown as dot plots, with one line per cell. Red dots indicate boundaries overlapping with the ground truth; non-overlapping boundaries are shown in gray. Results are based on 100-kb resolution data with 9× downsampling.

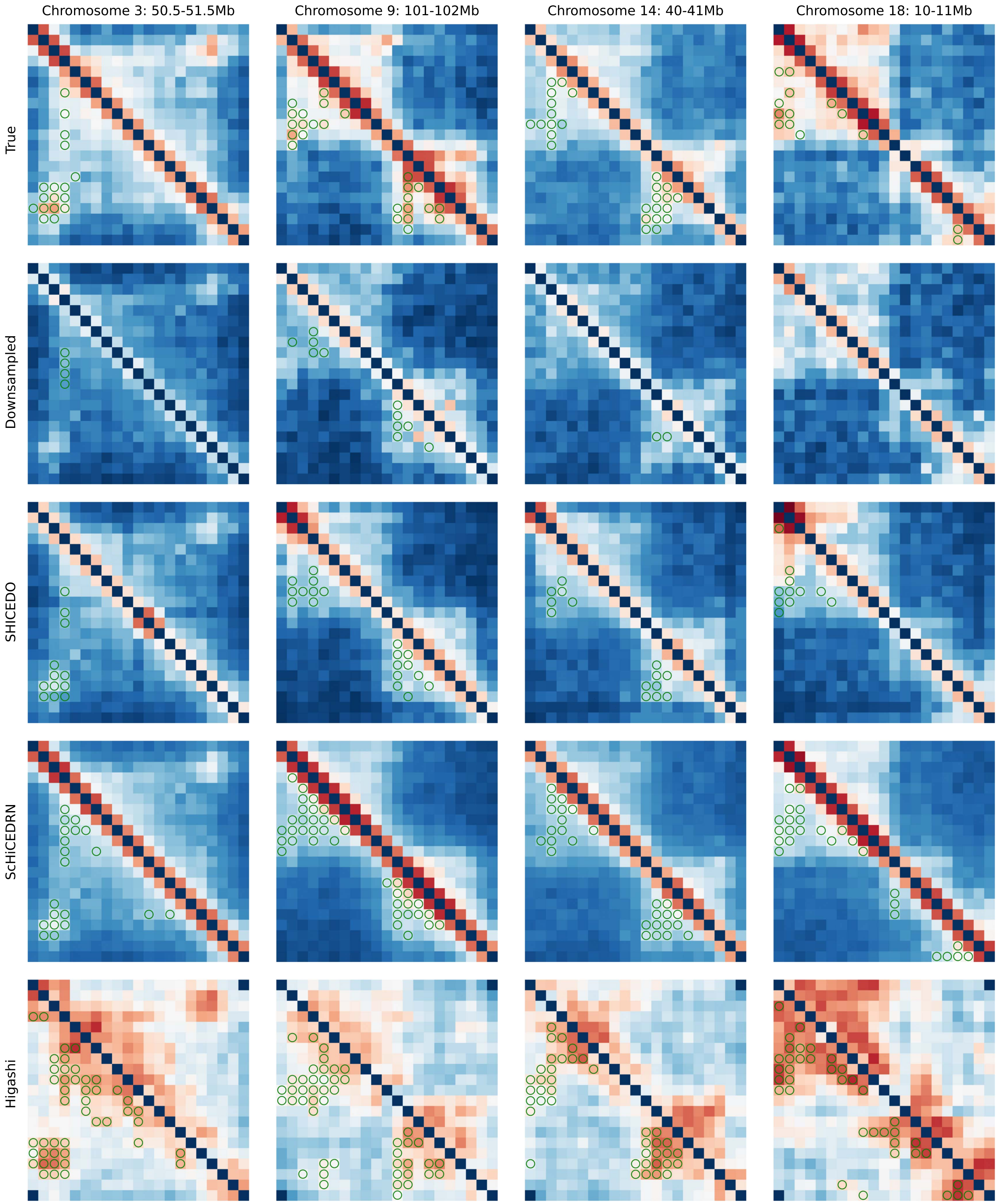


**Supplementary Figure S4: Visual illustration of chromatin loops detected in scHi-C data from the Lee *et al.* dataset.** Green circles indicate chromatin loops detected across 423 single cells in the test set. Heatmaps display the aggregated scHi-C contact matrices in four example regions: chromosome 3 (50.5– 51.5 Mb), chromosome 9 (101–102 Mb), chromosome 14 (40–41 Mb), and chromosome 18 (10–11 Mb). From top to bottom, the panels display the original high-coverage (True), downsampled sparse input (Downsampled), and enhanced matrices by SHICEDO, ScHiCEDRN, and Higashi. Results are based on 50-kb resolution data with 2× downsampling.

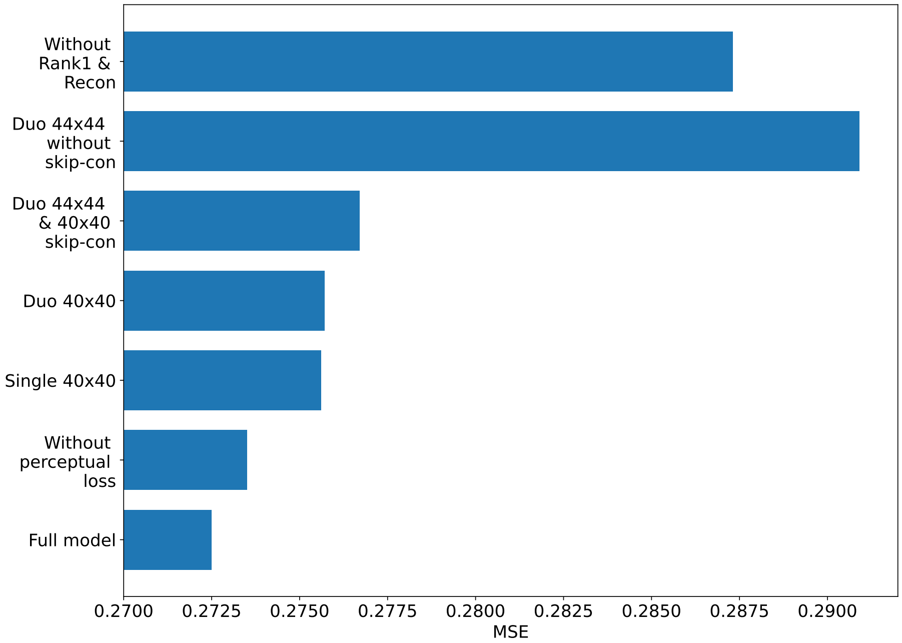


**Supplementary Figure S5**: **Ablation study - Performance variation across different model settings.** The bar plot presents the results derived from our ablation study, focusing on model design and loss function: the performance testing of MSE loss across single and dual branches with varying input sizes and skip connection strategies, the effectiveness of the rank-one feature extraction and reconstruction process, and an assessment of the perceptual loss. These evaluations were conducted on the Lee *et al.* dataset at 1-Mb resolution with a downsampling ratio of 16.

| **Dataset** | **GEO #** | **Matrix resolution** | **Data condition** | **# Cells** | **Average contacts per cell** |
|---|---|---|---|---|---|
| Lee *et al.* [2] | GSE130711 | 1 Mb | Raw | 4237 | 1,241,652 |
| | | | Cell-quality filter applied | 1058 | 1,958,026 |
| | | | Genomic-distance filter (1–20 Mb) applied | 1058 | 446,582 |
| | | | 36× downsampled | 1058 | 12,350 |
| | | 100 kb | Raw | 4237 | 1,241,652 |
| | | | Cell-quality filter applied | 1058 | 1,958,026 |
| | | | Genomic-distance filter (100 kb–2 Mb) applied | 1058 | 665,528 |
| | | | 9× downsampled | 1058 | 72,792 |
| | | 50 kb | Raw | 4237 | 1,241,652 |
| | | | Cell-quality filter applied | 1058 | 1,958,026 |
| | | | Genomic-distance filter (50 kb–1 Mb) applied | 1058 | 336,953 |
| | | | 2× downsampled | 1058 | 169,385 |
| Liu *et al.* [3] | GSE223917 | 1 Mb | Raw | 7469 | 277,542 |
| | | | Cell-quality filter applied | 4192 | 320,903 |
| | | | Genomic-distance filter (1–20 Mb) applied | 4192 | 146,035 |
| | | | 9× downsampled | 4192 | 16,257 |
| | | | 16× downsampled | 4192 | 9,147 |
| Nagano *et al.* [4] | GSE94489 | 1 Mb | Raw | 1171 | 294,726 |
| | | | Cell-quality filter applied | 464 | 400,813 |
| | | | Genomic-distance filter (1–20 Mb) applied | 464 | 98,381 |
| | | | 9× downsampled | 464 | 10,912 |
| Tan *et al.* [5] | GSE162511 | 1 Mb | Raw | 3646 | 365,076 |
| | | | Cell-quality filter applied | 3061 | 395,833 |
| | | | Genomic-distance filter (1–20 Mb) applied | 3061 | 125,239 |
| | | | 4× downsampled | 3061 | 31,269 |

**Supplementary Table S1**: **Summary for all scHi-C datasets used in this study.** The table summarizes the four scHi-C datasets, including their GEO accession numbers, preprocessing steps, the number of cells retained, and the average number of contacts per cell. The cell-quality filter removes low-quality cells based on minimum chromatin contact count thresholds. The genomic-distance filter excludes contacts beyond a specified genomic distance, removes diagonal contacts, and retains only autosomes and the X chromosome.

| **Evaluation task** | **Dataset (resolution, downsampling ratio)** | **Genomic-distance filtering** | **Bin-shift window** |
|---|---|---|---|
| Pixel-wise similarity & Hi-C-specific similarity (Deep learning methods) | Lee *et al.* (1-Mb resolution, 36× downsampling); Liu *et al.* (1-Mb resolution, 16× downsampling); Nagano *et al.* (1-Mb resolution, 9× downsampling); Tan *et al.* (1-Mb resolution, 4× downsampling) | 1–20 Mb | N/A |
| Pixel-wise similarity & Hi-C-specific similarity (Lightweight methods) | Liu *et al.* (1-Mb resolution, 16× downsampling); Liu *et al.* (1-Mb resolution, 9× downsampling) | 1–20 Mb | N/A |
| A/B compartment identification | Lee *et al.* (1-Mb resolution, 36× downsampling) | 1–20 Mb | 1–6 bins |
| TAD-like boundary detection | Lee *et al.* (100-kb resolution, 9×downsampling) | 100 kb–2 Mb | 1–6 bins |
| Chromatin-loop calling | Lee *et al.* (50-kb resolution, 4× downsampling) | 50 kb–1 Mb | 5 bins |

**Supplementary Table S2**: **Summary of datasets and evaluation settings used in this study.** This table lists the evaluation tasks, datasets, resolutions, downsampling ratios, genomic-distance filters, and bin-shift windows applied in different benchmarking experiments. Deep learning methods refer to neural network-based Hi-C enhancement models, including SHICEDO, ScHiCEDRN, Higashi, EnHiC, and DeepHiC. Lightweight methods refer to classical imputation approaches, including nearest-neighbor cell averaging, random walk with restart, and random forest–based strategies. Genomic-distance thresholds follow established best practices for different tasks: 20 Mb for analyses at 1-Mb resolution; 2 Mb for TAD detection at 100-kb resolution [6–8]; and 1 Mb for loop detection at 50-kb resolution [9]. Bin-shift-based matching thresholds also follow established practices: 1–6 bins for A/B compartment and TAD-like boundary evaluation [7, 10], and 5 bins for chromatin loop evaluation [9].

| | | 1 | 2 | 3 | 4 | 5 | 6 |
|---|---|---|---|---|---|---|---|
| Precision | Downsampled | 0.805 ± 0.169 | 0.8474 ± 0.1651 | 0.8685 ± 0.153 | 0.8818 ± 0.145 | 0.9019 ± 0.1305 | 0.9213 ± 0.1163 |
| | **SHICEDO** | **0.8467 ± 0.1389** | **0.8778 ± 0.1349** | **0.8945 ± 0.1229** | **0.9092 ± 0.1139** | **0.9326 ± 0.0879** | **0.9485 ± 0.0746** |
| | ScHiCEDRN | 0.8151 ± 0.1328 | 0.8679 ± 0.1285 | 0.8927 ± 0.1204 | 0.9109 ± 0.1085 | 0.9266 ± 0.0989 | 0.9416 ± 0.087 |
| | Higashi | 0.7463 ± 0.0783 | 0.8204 ± 0.0614 | 0.8676 ± 0.0626 | 0.9022 ± 0.0559 | 0.9291 ± 0.0407 | 0.9486 ± 0.031 |
| Recall | Downsampled | 0.8902 ± 0.0906 | 0.9256 ± 0.0681 | 0.9446 ± 0.0541 | 0.9574 ± 0.043 | 0.9661 ± 0.0359 | 0.9725 ± 0.0314 |
| | **SHICEDO** | **0.8806 ± 0.0928** | **0.93 ± 0.0642** | **0.9562 ± 0.0436** | **0.9724 ± 0.0297** | **0.9822 ± 0.0197** | **0.9884 ± 0.013** |
| | ScHiCEDRN | 0.8124 ± 0.1185 | 0.8755 ± 0.0994 | 0.9107 ± 0.0839 | 0.9332 ± 0.0719 | 0.9492 ± 0.0628 | 0.9604 ± 0.0562 |
| | Higashi | 0.7886 ± 0.1383 | 0.8425 ± 0.1184 | 0.8797 ± 0.1009 | 0.9066 ± 0.0864 | 0.9266 ± 0.0743 | 0.9419 ± 0.0636 |
| Macro F1 Score | Downsampled | 0.8695 ± 0.0797 | 0.9108 ± 0.0658 | 0.9348 ± 0.0541 | 0.9513 ± 0.0432 | 0.9634 ± 0.0345 | 0.9724 ± 0.028 |
| | **SHICEDO** | **0.8763 ± 0.0774** | **0.9225 ± 0.0577** | **0.9487 ± 0.042** | **0.9661 ± 0.0299** | **0.9774 ± 0.021** | **0.985 ± 0.0146** |
| | ScHiCEDRN | 0.8276 ± 0.0788 | 0.8876 ± 0.0633 | 0.9202 ± 0.053 | 0.9405 ± 0.0454 | 0.955 ± 0.0393 | 0.966 ± 0.0345 |
| | Higashi | 0.7912 ± 0.0824 | 0.8541 ± 0.0688 | 0.8949 ± 0.059 | 0.9224 ± 0.0516 | 0.9419 ± 0.0454 | 0.9558 ± 0.0397 |

**Supplementary Table S3**: **Precision, recall, and macro F1 score evaluations of single-cell A/B compartment identification from the Lee *et al.* dataset.** Performance metrics are reported for compartments identified from downsampled sparse input and enhanced scHi-C matrices, compared against the ground truth. Mean ± standard deviation values are calculated across cells, using different overlap thresholds ranging from 1 to 6 genomic bins. The highest values for each threshold are highlighted in blue; SHICEDO results are shown in bold. Results are based on 1-Mb resolution data with 36× downsampling.

| | | 1 | 2 | 3 | 4 | 5 | 6 |
|---|---|---|---|---|---|---|---|
| Precision | Downsampled | 0.5798 ± 0.0709 | 0.7181 ± 0.0374 | 0.8184 ± 0.0217 | 0.8873 ± 0.0164 | 0.9315 ± 0.0117 | 0.9590 ± 0.0086 |
| | **SHICEDO** | **0.5527 ± 0.0743** | **0.6908 ± 0.0475** | **0.7811 ± 0.0345** | **0.8440 ± 0.031** | **0.8875 ± 0.0284** | **0.9172 ± 0.0265** |
| | ScHiCEDRN | 0.5913 ± 0.0844 | 0.7252 ± 0.0543 | 0.8104 ± 0.0385 | 0.867 ± 0.0322 | 0.9048 ± 0.0273 | 0.93 ± 0.0241 |
| | Higashi | 0.5466 ± 0.0816 | 0.6647 ± 0.0992 | 0.7465 ± 0.1105 | 0.8025 ± 0.1173 | 0.8402 ± 0.1211 | 0.8652 ± 0.123 |
| Recall | Downsampled | 0.5033 ± 0.0781 | 0.6205 ± 0.0609 | 0.7037 ± 0.0541 | 0.7663 ± 0.052 | 0.8151 ± 0.0487 | 0.8534 ± 0.0459 |
| | **SHICEDO** | **0.6029 ± 0.0746** | **0.7560 ± 0.0432** | **0.8515 ± 0.0247** | **0.9129 ± 0.0166** | **0.9503 ± 0.0101** | **0.9723 ± 0.0064** |
| | ScHiCEDRN | 0.5668 ± 0.0762 | 0.7011 ± 0.0466 | 0.7956 ± 0.0306 | 0.8635 ± 0.0231 | 0.9114 ± 0.0156 | 0.9434 ± 0.0103 |
| | Higashi | 0.5819 ± 0.0275 | 0.7083 ± 0.035 | 0.7980 ± 0.0397 | 0.8612 ± 0.0428 | 0.9034 ± 0.0445 | 0.9304 ± 0.0452 |
| Macro F1 Score | Downsampled | 0.5415 ± 0.0733 | 0.6696 ± 0.0475 | 0.7615 ± 0.0369 | 0.8266 ± 0.0339 | 0.8722 ± 0.0308 | 0.9044 ± 0.0286 |
| | **SHICEDO** | **0.5778 ± 0.0741** | **0.723 ± 0.0445** | **0.8164 ± 0.0283** | **0.879 ± 0.0222** | **0.9198 ± 0.0178** | **0.9456 ± 0.0151** |
| | ScHiCEDRN | 0.5791 ± 0.0801 | 0.7120 ± 0.0502 | 0.8007 ± 0.0341 | 0.8627 ± 0.0272 | 0.9058 ± 0.021 | 0.9349 ± 0.0169 |
| | Higashi | 0.5642 ± 0.0524 | 0.6864 ± 0.0643 | 0.7719 ± 0.0721 | 0.8314 ± 0.0771 | 0.8714 ± 0.0801 | 0.8976 ± 0.0816 |

**Supplementary Table S4**: **Precision, recall, and macro F1 score evaluations of single-cell TAD-like domain detection from the Lee *et al.* dataset.** Performance metrics are reported for TAD-like domain boundaries detected from downsampled sparse input and enhanced scHi-C matrices, compared against the ground truth. Mean ± standard deviation values are calculated across cells, using different overlap thresholds ranging from 1 to 6 genomic bins. The highest values for each threshold are highlighted in blue; SHICEDO results are shown in bold. Results are based on 100-kb resolution data with 9× downsampling.

| Method | Training time per epoch | Peak GPU memory (MiB) |
|---|---|---|
| SHICEDO | 0:45:16 | 24,013 |
| ScHiCEDRN | 1:24:08 | 24,710 |
| Higashi | 0:00:51 | 2,032 |
| EnHiC | 0:13:01 | 79,649 |
| DeepHiC | 0:03:01 | 3,908 |

**Supplementary Table S5: Computational cost analysis of deep-learning methods on the Liu *et al.* dataset.** Training was performed on an NVIDIA A100 (80 GB) GPU paired with an AMD EPYC 7543 32-core CPU. Values show the wall-clock training time per epoch and the peak GPU memory consumption for each method. Results are based on 1-Mb resolution data with 16× downsampling. Batch size = 128.

| **Downsampling** | **Method** | **MAE ↓** | **Macro F1 ↑** | **HiCRep SCC ↑** | **GenomeDISCO ↑** |
|---|---|---|---|---|---|
| 9× | Downsampled | 0.3826 | 0.6955 | 0.7769 | 0.4845 |
| | **SHICEDO** | **0.2814** | **0.7377** | **0.8013** | **0.6611** |
| | KNN5 | 0.4501 | 0.6182 | 0.3974 | 0.5576 |
| | RF | 0.8687 | 0.3118 | 0.1410 | 0.4230 |
| | RWR | 0.4782 | 0.3811 | 0.7317 | −0.7054 |
| 16× | Downsampled | 0.4288 | 0.6167 | 0.6731 | 0.1994 |
| | **SHICEDO** | **0.0986** | **0.8797** | **0.9159** | **0.9196** |
| | KNN5 | 0.4670 | 0.6188 | 0.3656 | 0.4902 |
| | RF | 0.8432 | 0.3273 | 0.1566 | 0.4032 |
| | RWR | 0.4807 | 0.4629 | 0.6627 | −0.7031 |

**Supplementary Table S6**: **Pixel-wise and Hi-C-specific similarity evaluation of enhanced scHi-C data using Liu *et al.* dataset.** In this evaluation, SHICEDO is compared with three lightweight baselines: KNN5, which imputes each contact map by averaging the five nearest neighboring cells; RF, a distance-aware random forest imputation; and RWR, a random walk with restart method. Two pixel-wise metrics, mean absolute error (MAE) and macro F1 score, were computed by comparing the downsampled sparse input and enhanced scHi-C sub-matrices against the ground truth. Additionally, two Hi-C–specific similarity measures, HiCRep SCC and the GenomeDISCO scores, were calculated from chromosome-wide matrices. Arrows in the column headers indicate the desired direction of improvement for each evaluation metric. The highest values for each threshold are highlighted in blue; SHICEDO results are shown in bold. Results are based on 1-Mb resolution data with 9× and 16× downsampling.

# 2 Supplementary Notes

## 2.1 The GAN architecture in SHICEDO

SHICEDO is built on a generative adversarial network (GAN) framework and consists of two key components: the generator (Enhancement Network) and the discriminator (Discriminator Network), as illustrated in Figure 1 in the main text.

The generator adopts a multi-branch architecture, allowing the input of submatrices with different sizes (i.e. dimensionality of matrices) or resolutions (i.e., genomic bin sizes). Each generator branch comprises three key components: a rank-one feature extraction module, a reconstruction module, and a feature refinement module. Output submatrices from all branches are merged and further refined through two additional feature refinement modules to produce a final enhanced output.

The discriminator comprises a rank-one feature extraction module, a reconstruction module, two convolutions and Squeeze-and-Excitation (SE) modules, and a fully connected layer applied after flattening the intermediate representations to produce predictions.

To optimize feature extraction and selection for single-cell Hi-C (scHi-C) data, our SE module incorporates a channel-wise attention mechanism, as inspired by Squeeze-and-Excitation Networks [11]. Additionally, skip connections are employed throughout the network to minimize information loss.

**Abbreviations, symbols, and terms used in Figure 1:**

**$\times$:** Outer product across the last two spatial axes
**$+$:** Element-wise sum of two tensors
**$||$:** Concatenation of two tensors
**V:** Rank-one feature
**$\mathbf{V}^{\mathsf{T}}$:** Transpose of the rank-one feature
**SE:** Squeeze-and-Excitation network
**FFN:** Classification head consisting of flattening followed by a fully connected layer
**Input:** A normalized downsampled 40×40 scHi-C submatrix and a 44×44 submatrix that includes a two-bin border around the 40×40 central region
**Ground Truth:** A 40×40 submatrix extracted from the original non-downsampled, normalized scHi-C contact matrix
**Output:** The model-enhanced 40×40 scHi-C submatrix

## 2.2 Representation of scHi-C data in the SHICEDO model

**Representation of scHi-C contact frequency matrices** We begin by introducing notations for the scHi-C contact frequency matrices. In a scHi-C experiment, chromatin contact profiles are measured at the level of individual cells. For each cell $k$, the contact frequency matrix is represented as a non-negative, symmetric, and typically sparse matrix of size $N \times N$, where $N$ denotes the number of non-overlapping genomic bins at a fixed resolution. Each entry in the matrix, denoted as $C_{ij}^{k}$ indicates the observed contact frequency between genomic loci $i$ and $j$ in cell $k$; higher values correspond to closer spatial proximity between the loci pair within the cell nucleus.

In this work, we focus only on intra-chromosomal contact matrices. After preprocessing and normalization (see Supplementary Note 2.9 for details), the scHi-C data were randomly divided by cell into a training set, validation set, and test set, comprising approximately 70%, 10%, and 20% of the total cells, respectively. For the single-cell loop calling task, the split was adjusted to 55%, 5%, and 40% to ensure that the test set contained a sufficient number of cells for SnapHiC [9] loop detection.

**scHi-C tensor data** Because full chromosome-wide scHi-C contact matrices are too large for direct GPU processing, we adopt a patch-based strategy that is widely used in Hi-C enhancement studies [6, 7, 12–14]. Specifically, we extract overlapping square patches of size $n$ by $n$ (e.g., $40\times40$ submatrices) from each intra-chromosomal matrix. Each patch is treated as an independent data sample and used as input to the model.

The scHi-C data is fed to the network as a tensor of shape (batch, 1, $n$, $n$), where the single channel encodes the contact intensity values.

**Channels in the SHICEDO network** The input tensor begins with a single channel representing normalized contact frequencies. After the data passes through initial convolutional layers of the network, the channel dimension is expanded (e.g., $1 \to 64 \to 128 \to \cdots$) to learn and store intermediate feature representations. The final decoder then collapses this expanded multi-channel representation back into a single channel, yielding the enhanced scHi-C contact map.

### 2.3 Rank-one feature extraction: concept and implementation

Traditional one-dimensional (1D) convolutional layers typically operate with small kernels (e.g., $1\times k$ or $k\times 1$) that slide along a single spatial axis. While effective in many applications, such convolutions cannot fully capture the two-dimensional (2D) structure of Hi-C contact maps, especially in the context of sparse scHi-C data.

To overcome this limitation, SHICEDO employs a rank-one block [7], a specialized module designed to extract low-rank features while preserving the symmetric and non-negative properties of scHi-C data. Instead of relying on local sliding kernels, our rank-one block reduces dimensionality along one axis and reconstructs a full matrix through an outer product, enabling explicit modeling of global patterns.

Specifically, given an input tensor of shape (batch, 1, $n$, $n$), the rank-one block applies $C$ learnable $1\times n$ kernels to each submatrix, summarizing contact intensities along each row. This produces s a tensor $\mathbf{V}$ of shape (batch, $C$, $n$, 1), where $C$ is the number of channels. The transpose $\mathbf{V}^{\mathsf{T}}$ with shape (batch, $C$, 1, $n$) is then computed. The outer product $\mathbf{V}\times\mathbf{V}^{\top}$ yields a reconstructed tensor of shape (batch, $C$, $n$, $n$), in which each channel represents a rank-one symmetric, non-negative matrix.

Here, the rank-one feature refers to the intermediate narrow column-vector of shape (batch, $C$, $n$, 1). Each reconstructed matrix channel is, by definition, rank-one, and captures broad, multiplicative row-by-column variation. These rank-one structures encode global trends in the contact map, allowing the network to isolate and discount low-frequency signals. This enhances the model's ability to detect finer-scale, biologically meaningful chromatin interaction features embedded in the residual signal; such features that might be overlooked by standard convolutional layers.

Thus, the rank-one block provides an explicit low-rank factorization step that can not be achieved by conventional 1D convolutions, offering both interpretability and improved effectiveness for scHi-C data enhancement.

## 2.4 Module descriptions in the SHICEDO model

This section details the core components of the SHICEDO architecture, including the feature extraction, reconstruction, refinement, and attention modules used in both the generator and discriminator networks.

**Rank-one feature extraction and reconstruction modules** The rank-one feature extraction module is designed to capture features that may be overlooked by traditional 2D convolutions. It employs a 1D kernel with parameters shared across genomic bins. This module comprises a rank-one convolutional layer followed by two additional 1D convolutional layers. Between these layers, squeeze-and-extraction (SE) modules and ReLU activation functions are integrated, with skip connections applied to facilitate tensor flow. Subsequently, a weight module applies a set of learnable weights to the tensor. Both the resultant rank-one feature tensor and its transpose tensor are further refined through two 1D SE modules before entering the feature reconstruction module. The feature reconstruction module is designed to reshape tensors to preserve the symmetric property that is characteristic of scHi-C data. The outer product of the rank-one feature tensors is dire a 2D SE module.

**Feature refinement module** The feature refinement module is designed to further enhance the intricate structural details within the tensor after its reconstruction. It encompasses two 2D convolutional layers. After each of these layers, SE modules and a ReLU activation function are incorporated. Additionally, spectral normalization is applied to parameters within each 2D convolutional layer.

**Squeeze-and-extraction (SE) module** The SE module is crafted to improve the selection of features along the channel dimension within internal tensors. The SE module begins with an average pooling layer, followed by a fully connected layer and a ReLU activation function. Subsequently, another fully connected layer is employed, followed by a Sigmoid activation function to ensure the learnable weights range between 0 and 1. Ultimately, these channel-wise weights are multiplied with the input feature tensor. There are two types of SE modules: the 1D SE module aids in the channel-wise feature selection of rank one features tensors, while the 2D SE module is applied to the internal tensors post-reconstruction. Although their functions are analogous, they are tailored for distinct dimensional operations.

### 2.5 Loss function

The loss function used by the generator in SHICEDO contains five terms, as expressed in Equation 1, with parameters $w_1$ to $w_5$ that require fine-tuning. The five core components include binary cross-entropy (BCE) for adversarial (Adv) loss (Equation 2), structural dissimilarity (DSSIM) loss [15] (Equation 3), mean square error (MSE) loss, customized perceptual loss (PL) [16], and feature matching (FM) loss. Each of these components plays a specific role in evaluating the generator's performance. The MSE loss and BCE loss are primarily responsible for high-level performance evaluations. The MSE loss conducts an overarching pixel-wise assessment of the entire matrix, while the BCE loss reflects the discriminator's cumulative appraisal of the qualify of predicted scHi-C matrices. The DSSIM loss evaluates local matrix structures, contributing to the evaluation of the Hi-C matrix from a structural perspective. The perceptual loss measures the difference between high-level features extracted from pre-trained convolutional networks, aiming to make the generated matrix perceptually similar to the target matrix rather than merely minimizing pixel-wise differences. In particular, we developed a scHi-C denoising model employing a U-net architecture [17] optimized for perceptual loss. The feature matching loss applies discriminator-extracted features to assess Hi-C features. Collectively, these loss components combine pixel-wise, structural, and feature evaluations to ensure the enhanced scHi-C matrix is both faithful to the original data and perceptually similar to it.

$$\text{Loss}_{Gen} = w_1(L_{\text{Adv}}) + w_2(L_{\text{MSE}}) + w_3(L_{\text{DSSIM}}) + w_4(L_{\text{PL}}) + w_5(L_{\text{FM}}) \tag{1}$$

$$\text{Loss}_{Adv} = L_{\text{BCE}}(1, \text{Dis}(\text{Gen}(M_{\text{LR}}))) \tag{2}$$

$$\text{Loss}_{\text{DSSIM}} = \frac{1 - \text{SSIM}}{2} \in [0, 1] \tag{3}$$

The discriminator's loss function is represented in Equation 4.

$$\text{Loss}_{Dis} = L_{\text{BCE}}(1, \text{Dis}(M_{\text{True}})) + L_{\text{BCE}}(0, \text{Dis}(\text{Gen}(M_{\text{LR}}))) \tag{4}$$

**Details of the modules in U-net for perceptual loss and its implementation** Perceptual loss is a specialized loss function designed to evaluate the dissimilarity between high-level features extracted from pre-trained convolutional networks. Its objective is to make the generated matrix perceptually similar to the target matrix, rather than solely minimizing pixel-wise discrepancies. By integrating both pixel-wise and perceptual loss terms into the generator's loss function, a more nuanced matrix generation is achieved, ensuring that the generated matrix is closely aligned with the target not only in terms of pixel distribution but also in high-level feature consistency.

Our scHi-C denoising model employs a U-net [17] architecture optimized for perceptual loss. The encoder comprises three convolutional blocks, each consisting of a 2D convolutional layer, normalization layers, a squeeze-and-excitation (SE) module [11], and activation layers. The decoder is symmetrically configured with transposed convolutional layers, normalization layers, SE modules, and activation layers. The use of skip connections facilitates information

flow between the encoder and decoder modules. The SE module configurations are congruent with those deployed in the SHICEDO model.
To introduce noise, we conducted stochastic imputation using a random walk algorithm as implemented in scHiCTools [18]. The model was trained on 70% of the cells from the 1-Mb resolution Nagano *et al.* dataset, with 10% of the data allocated for validation and the remaining 20% for testing.
Regarding the perceptual loss, a tunable parameter was introduced to specify the number of encoder blocks contributing to loss computation. Depending on the experimental setting, this parameter requires optimization. In the current study, all three encoder blocks were engaged in calculating the perceptual loss.

**Feature matching loss and DSSIM loss** Within our computational framework, feature matching loss is quantified using the mean absolute error (MAE) to measure the difference between the actual and predicted rank-one module output tensors, as well as the output tensors of two SE modules after the reconstruction module in the discriminator network. Additionally, we employ the DSSIM loss, a derivative of the structural similarity index (SSIM), defined in Equation 3 [7].

**Adversarial loss** Binary cross-entropy (BCE) loss serves as an additional metric, computing the binary cross entropy between the provided labels and their corresponding inputs. The adversarial loss for the generator is determined by calculating BCE between the enhanced matrix and a reference matrix comprising solely of unit elements, as shown in Equation 2. A conventional adversarial loss function is implemented for the discriminator. This function is configured to prompt the discriminator to yield predictions approximating one for ground-truth matrices, and values nearing zero for model-enhanced matrices, as delineated in Equation 4.

### 2.6 Multi-size vs. Multi-scale input design

The SHICEDO model architecture supports parallel input branches, allowing flexibility in how contextual information is integrated during training and inference. The generator can process submatrices of different sizes (i.e., dimensionality of matrices) or different resolutions (i.e., genomic bin sizes).

**Multi-size input (current implementation):** In this study, SHICEDO employs a dual-branch configuration, accepting input submatrices with the same resolution (i.e., genomic bin size) but two different dimensions: one branch processes a $40 \times 40$ scHi-C submatrix, while the other takes a slightly larger $44 \times 44$ submatrix that fully contains the $40 \times 40$ central region plus a two-bin border on all sides. The larger submatrix provides additional contextual and boundary information, which helps mitigate edge artifacts during the enhancement of the central $40 \times 40$ region. This dual-size configuration has demonstrated strong performance (see Supplementary Note 2.8 for the ablation study) and is adopted throughout our analyses.

**Multi-scale input (architectural capability):** SHICEDO's architecture also supports a multi-scale input design, in which submatrices at different resolutions (e.g., genomic bin sizes of 50 kb and 100 kb) can be provided. This configuration would allow the model to learn both fine-grained and coarse-grained chromatin interaction simultaneously. Although this multi-scale strategy is not employed in the present work, we consider it a promising direction for future model development (see Discussion section in the main text).

### 2.7 Important parameters of SHICEDO

In addition to standard hyperparameters such as learning rate and batch size, which require fine-tuning in the SHICEDO framework, several key parameters warrant additional attention. These parameters include the channel numbers for convolutional layers, the reduction coefficient for squeeze-and-excitation (SE) modules, the weighting parameters for the generator's loss function, and the selection of encoder blocks for perceptual loss computation. Given the sensitivity of GANs to parameter choices [19], improper configurations, such as suboptimal channel numbers, reduction parameters, or learning rates, can induce model collapse. The parameters that were found to work well in our experiments are provided in our GitHub repository (`https://github.com/wmalab/SHICEDO`).

The variability in input matrix dimensions necessitates corresponding adjustments in the path numbers and padding parameters for the rank-one module within the generator. In our current implementation, we utilize dual branches for input matrices sized at 40×40 and 44×44, as guided by our ablation study (Supplementary Note 2.8). Although multi-scale approaches are also viable, they would require careful tensor integration to ensure compatibility.

### 2.8 Ablation study

In this section, we conducted an ablation study to thoroughly investigate the functions and effectiveness of each component within our model. The components under examination include the multi-input pathway, the rank-one feature extraction and reconstruction module, the channel-wise attention module (i.e., the SE module), and our specially designed perceptual loss function (Supplementary Figure S5).

Our exploration began with the rank-one feature extraction and reconstruction module. We substituted it with a traditional convolution residual block to discern its contribution to overall performance. The resultant data revealed a significant improvement in performance due to the rank-one module. We hypothesize that this improved performance stems from the rank-one module's capability to extract features that might be overlooked by traditional CNNs and induce symmetry in latent features, aligning with the intrinsic feature architecture of Hi-C matrices.

Next, we assessed the importance of the channel-wise attention (SE) module, which plays a crucial role in feature selection and overall model stability. Upon removing the SE module, the model could not produce any meaningful predictions without significant modifications to the remaining components. To maintain a fair comparison across the different modules, other components were held constant.

Furthermore, we tested the flexibility of our model with multi-size input branches. Experimental results indicated the model achieved optimal performance when provided with one input of the desired size and another slightly larger. The model delivered superior performance when the inputs were $40\times40$ and $44\times44$ submatrices. As outlined in the future directions (the Discussion section in the main text), this flexible multi-path design equips our model to handle multi-size or multi-scale inputs effectively.

Lastly, to evaluate the efficacy of our perceptual loss term within the loss function, we utilized the 1-Mb resolution Nagano *et al.* dataset to pre-train our custom U-net optimized for perceptual loss. Then, we performed the enhancement task on the Lee *et al.* dataset. Such a scenario exemplifies conditions in which suitable pre-training data might be scarce or when computational resources are restrictive, rendering model pre-training impractical with each new dataset deployment. Notably, our findings highlight that, despite potential discrepancies in sequencing depths between the training datasets, the perceptual loss model consistently bolsters overall performance.

### 2.9 Data availability and preprocessing

We applied SHICEDO to four scHi-C datasets: the human brain prefrontal cortex cells from Lee *et al.* [2] (GEO: GSE130711), the developing mouse embryonic cells from Liu *et al.* [3] (GEO: GSE223917), the mouse embryonic stem (ES) cells from Nagano *et al.* [4] (GEO: GSE94489), and the developing mouse brain cells from Tan *et al.* [5] (GEO: GSE162511) (Supplementary Table S1).
Starting from raw scHi-C data, we first applied a cell-quality filter, discarding any cell whose total contact counts fell below dataset-specific thresholds (see details below). Next, we applied a genomic-distance filter: diagonal entries were set to zero, only intra-chromosomal contacts within the specified genomic-distance thresholds were retained, and we restricted the analysis to autosomes and the X chromosome. The filtered matrices were then downsampled to the designated downsampling ratio. Finally, the downsampled scHi-C matrices were normalized and partitioned into fixed-size submatrices, which served as input samples for the enhancement models.

**Cell-quality filter** During preprocessing, we prioritized the quality of input data by applying dataset-specific thresholds on the minimum number of chromatin contacts per cell. For the Lee *et al.* dataset, we retained only cells with more than 760,000 contacts, yielding 1,058 cells spanning all 14 cell types. For the Liu *et al.* dataset, we used a threshold of 210,000 contacts, resulting in 4,192 cells after filtering. For the Nagano *et al.* dataset, the threshold was 310,000 contacts, producing 464 cells spanning all cellular stages. For the Tan *et al.* dataset, we applied a threshold of 210,000 contacts, retaining 3,061 cells covering all four main cell types and seven developmental stages. The detailed cell-quality filtering statistics are provided in Supplementary Table S1.

**ScHi-C matrix resolution** We processed the Lee *et al.* dataset at three different matrix resolutions: 1 Mb, 100 kb, and 50 kb. The other three datasets were processed at 1 Mb resolution. For downstream analyses, 1-Mb resolution data were used for pixel-wise and Hi-C-specific similarity evaluation as well as A/B compartment identification; 100-kb resolution data were used for TAD-like boundary detection; and 50-kb resolution data were used for chromatin loop calling (Supplementary Tables S1 and S2).

**Genomic-distance filter** For each intra-chromosomal scHi-C matrix, we focused our analyses on chromatin contacts within a specified genomic distance to retain informative contacts while reducing noise. At 1-Mb resolution, we applied a 20 Mb genomic-distance threshold. Chromatin contacts beyond this distance are generally too sparse and noisy to be informative, and their removal also improves computational efficiency. Specifically, we retained ∼95% of all intra-chromosomal contacts by applying such threshold in the Lee *et al.* dataset. For 100-kb resolution matrices used in TAD-like domain detection, we applied a 2 Mb threshold, given the observation that the average TAD size is around 1 Mb. This genomic-distance threshold is consistent with the settings used in hicGAN [6] and EnHiC [7]. For 50-kb resolution matrices used in chromatin loop detection, we applied a 1 Mb threshold, following the recommendation of SnapHiC [9]. In all cases, the main diagonal was removed to eliminate short-range contacts, which are predominantly artifacts. The detailed summary of the

genomic-distance thresholds and corresponding statistics is provided in Supplementary Table S1.

**Downsampling** To generate sparse input matrices, we implemented a random sampling procedure that was previously used in our earlier work, EnHiC [7]. For example, a downsampling ratio of 9 indicates that the contact count of the resulting scHi-C matrix is reduced to 1/9th of the original data.

**Data normalization** To mitigate the inherent biases in scHi-C data, we executed a two-step normalization procedure using BandNorm [20] followed by log normalization. It is important to note that, after the enhancement process, we reversed the log normalization before conducting evaluations and applications.

**Matrix partition** Post-normalization, each scHi-C matrix was partitioned into 40×40 submatrices along the diagonal with step size 20. We also included the optional input, the 44×44 submatrix paired with each 40×40 submatrix, capturing the same genomic region but with additional border pixels. For the initial and terminal 44×44 submatrices, we applied a two-layer zero padding along the border. When reconstructing the whole matrix from enhanced submatrices, we average the values of overlapping pixels.

## 2.10 Training and prediction

We implemented SHICEDO using PyTorch 2.0.1 [21], Python 3.9 and CUDA 11.7. Training was primarily performed on NVIDIA A100 and RTX3090Ti GPUs with a memory capacity of 128 GB.

Given that scHi-C datasets differ in resolution and downsampling ratios, the parameter settings and corresponding training durations varied across experiments. Currently, the network processes fixed 40×40 patches, so peak GPU memory depends only on model size and batch size, not dataset size. Training runtime scales linearly with the number of training patches and epochs.

For example, training on the Lee *et al.* dataset (1-Mb resolution, 36× downsampling, 1058 cells) for 200 epochs took ∼15 GPU hours on a single NVIDIA A100 (80GB GPU memory) with a batch size of 128. Under this configuration, the generator and discriminator contained 34,079,757 and 2,128,896 trainable parameters, respectively. Even for larger datasets, peak GPU memory remains determined by the model size and batch size, not by the number of cells. Training runtime increases roughly linearly with the total number of training patches. Inference, by contrast, is inexpensive: once the model is trained, the model only requires a forward pass to predict enhanced contact maps for additional cells, which is much faster than training and uses minimal GPU memory.

The computational cost of SHICEDO and other deep learning baselines is summarized in Supplementary Table S5. Because training time and peak GPU memory depend on batch size, number of epochs, and dataset size, we standardized the comparison using the Liu *et al.* dataset (1-Mb resolution, 16× downsampling, 4192 cells), with a fixed batch size of 128. Under these settings, Supplementary Table S5 reports the wall-clock training time per epoch and peak GPU memory consumption for each method.

Detailed configurations for all datasets are available in our GitHub repository (`https://github.com/wmalab/SHICEDO`). For evaluation and downstream applications, all baseline models were retrained on the respective datasets following the guidelines provided in the README files on the GitHub page.

### 2.11 Pixel-wise and Hi-C-specific similarity evaluation of lightweight methods

To evaluate whether our deep-learning model SHICEDO provides advantages beyond classic lightweight imputation methods, we compared it against three lightweight baselines: five-nearest-neighbor cell averaging (KNN5), distance-aware random-forest imputation (RF) [22], and random walk with restart (RWR) [23]. These methods are widely used because of their simplicity and low computational cost, but their effectiveness for extremely sparse scHi-C data remains uncertain. We performed the comparison on the Liu *et al.* dataset at 1-Mb resolution, with two downsampling ratios: $9\times$ and $16\times$. Evaluation included both pixel-wise accuracy metrics (MAE and macro F1 score) and Hi-C–specific structural similarity metrics (HiCRep SCC and GenomeDISCO).

As shown in Supplementary Table S6, SHICEDO achieved the lowest MAE, the highest macro F1 score, and the strongest HiCRep SCC and GenomeDISCO scores across both sparsity settings. These results confirmed that SHICEDO can recover fine-grained contacts while preserving large-scale chromatin architecture.

The lightweight baselines showed mixed and context-dependent performance. KNN5 was the most effective among them, since averaging each contact with those from five structurally similar cells consistently lifted the GenomeDISCO score at both $9\times$ and $16\times$ downsampling ratios, and improved macro F1 score on the very sparse $16\times$ downsampled data. RF provided limited benefit under extreme sparsity ($16\times$ downsampling), where its distance-aware prediction partially restored missing contacts; however, at $9\times$ downsampling, , the same mechanism tended to over-predict contacts and degraded all metrics. RWR performed poorly at both sparsity levels. This is because when the graph is already severely under-connected, as in scHi-C, repeated random walks amplified noise rather than revealing true structure, leading to higher pixel-wise errors and lower structural similarity values.

These observations illustrate that lightweight imputation methods, which leverage neighboring contacts or similar cells, can sometimes increase structural similarity metrics by boosting overall graph connectivity, which the GenomeDISCO metric rewards. However, such smoothing often reduces pixel-level accuracy and blurs the fine-scale chromatin features, leading to lower HiCRep SCC and macro F1 scores. In contrast, SHICEDO, built on advanced deep learning techniques, reliably enhances local contact patterns while simultaneously reinforcing global chromatin structures under both moderate ($9\times$) and extreme ($16\times$) sparsity conditions.

## Supplementary References


[1] Kai Kruse, Clemens B Hug, and Juan M Vaquerizas. FAN-C: a feature-rich framework for the analysis and visualisation of chromosome conformation capture data. *Genome biology*, 21(1):1–19, 2020.

[2] Dong-Sung Lee, Chongyuan Luo, Jingtian Zhou, Sahaana Chandran, Angeline Rivkin, Anna Bartlett, Joseph R Nery, Conor Fitzpatrick, Carolyn O'Connor, Jesse R Dixon, et al. Simultaneous profiling of 3D genome structure and DNA methylation in single human cells. *Nature methods*, 16(10):999–1006, 2019.

[3] Zhiyuan Liu, Yujie Chen, Qimin Xia, Menghan Liu, Heming Xu, Yi Chi, Yujing Deng, and Dong Xing. Linking genome structures to functions by simultaneous single-cell Hi-C and RNA-seq. *Science*, 380(6649):1070–1076, 2023.

[4] Takashi Nagano, Yaniv Lubling, Csilla Várnai, Carmel Dudley, Wing Leung, Yael Baran, Netta Mendelson Cohen, Steven Wingett, Peter Fraser, and Amos Tanay. Cell-cycle dynamics of chromosomal organization at single-cell resolution. *Nature*, 547(7661):61–67, 2017.

[5] Longzhi Tan, Wenping Ma, Honggui Wu, Yinghui Zheng, Dong Xing, Ritchie Chen, Xiang Li, Nicholas Daley, Karl Deisseroth, and X Sunney Xie. Changes in genome architecture and transcriptional dynamics progress independently of sensory experience during post-natal brain development. *Cell*, 184(3):741–758, 2021.

[6] Qiao Liu, Hairong Lv, and Rui Jiang. hicGAN infers super resolution Hi-C data with generative adversarial networks. *Bioinformatics*, 35(14):i99–i107, 2019.

[7] Yangyang Hu and Wenxiu Ma. EnHiC: learning fine-resolution Hi-C contact maps using a generative adversarial framework. *Bioinformatics*, 37(Supplement_1):i272–i279, 2021.

[8] Jingxuan Xu, Xiang Xu, Dandan Huang, Yawen Luo, Lin Lin, Xuemei Bai, Yang Zheng, Qian Yang, Yu Cheng, An Huang, et al. A comprehensive benchmarking with interpretation and operational guidance for the hierarchy of topologically associating domains. *Nature Communications*, 15(1):4376, 2024.

[9] Miao Yu, Armen Abnousi, Yanxiao Zhang, Guoqiang Li, Lindsay Lee, Ziyin Chen, Rongxin Fang, Taylor M Lagler, Yuchen Yang, Jia Wen, et al. SnapHiC: a computational pipeline to identify chromatin loops from single-cell Hi-C data. *Nature methods*, 18(9):1056–1059, 2021.

[10] Ruochi Zhang, Tianming Zhou, and Jian Ma. Multiscale and integrative single-cell Hi-C analysis with Higashi. *Nature biotechnology*, 40(2):254–261, 2022.

[11] Jie Hu, Li Shen, and Gang Sun. Squeeze-and-excitation networks. In *Proceedings of the IEEE conference on computer vision and pattern recognition*, pages 7132–7141, 2018.

[12] Y Zhang, L An, J Xu, B Zhang, WJ Zheng, M Hu, J Tang, and F Yue. Enhancing Hi-C data resolution with deep convolutional neural network HiCPlus. nat. commun. 9, 750, 2018.

[13] Hao Hong, Shuai Jiang, Hao Li, Guifang Du, Yu Sun, Huan Tao, Cheng Quan, Chenghui Zhao, Ruijiang Li, Wanying Li, et al. DeepHiC: A generative adversarial network for enhancing Hi-C data resolution. *PLoS computational biology*, 16(2):e1007287, 2020.

[14] Yanli Wang, Zhiye Guo, and Jianlin Cheng. Single-cell Hi-C data enhancement with deep residual and generative adversarial networks. *Bioinformatics*, page btad458, 2023.

[15] Zhou Wang, Alan C Bovik, Hamid R Sheikh, and Eero P Simoncelli. Image quality assessment: from error visibility to structural similarity. *IEEE transactions on image processing*, 13(4):600–612, 2004.

[16] Tim Salimans, Ian Goodfellow, Wojciech Zaremba, Vicki Cheung, Alec Radford, and Xi Chen. Improved techniques for training GANs. *Advances in neural information processing systems*, 29, 2016.

[17] Olaf Ronneberger, Philipp Fischer, and Thomas Brox. U-net: Convolutional networks for biomedical image segmentation. In *Medical Image Computing and Computer-Assisted Intervention–MICCAI 2015: 18th International Conference, Munich, Germany, October 5-9, 2015, Proceedings, Part III 18*, pages 234–241. Springer, 2015.

[18] Xinjun Li, Fan Feng, Hongxi Pu, Wai Yan Leung, and Jie Liu. scHiCTools: A computational toolbox for analyzing single-cell Hi-C data. *PLoS computational biology*, 17(5):e1008978, 2021.

[19] Ian Goodfellow, Jean Pouget-Abadie, Mehdi Mirza, Bing Xu, David Warde-Farley, Sherjil Ozair, Aaron Courville, and Yoshua Bengio. Generative adversarial nets. *Advances in neural information processing systems*, 27, 2014.

[20] Ye Zheng, Siqi Shen, and Sündüz Keleş. Normalization and de-noising of single-cell Hi-C data with BandNorm and scVI-3D. *Genome Biology*, 23(1):222, 2022.

[21] Adam Paszke, Sam Gross, Francisco Massa, Adam Lerer, James Bradbury, Gregory Chanan, Trevor Killeen, Zeming Lin, Natalia Gimelshein, Luca Antiga, et al. Pytorch: An imperative style, high-performance deep learning library. *Advances in neural information processing systems*, 32, 2019.

[22] My Nguyen, Brydon PG Wall, J Chuck Harrell, and Mikhail G Dozmorov. scHiCcompare: an R package for differential analysis of single-cell Hi-C data. *Journal of Molecular Biology*, page 169155, 2025.

[23] Jingtian Zhou, Jianzhu Ma, Yusi Chen, Chuankai Cheng, Bokan Bao, Jian Peng, Terrence J Sejnowski, Jesse R Dixon, and Joseph R Ecker. Robust single-cell Hi-C clustering by convolution-and random-walk–based imputation. *Proceedings of the National Academy of Sciences*, 116(28):14011–14018, 2019.
